# A Complex Extracellular Sphingomyelinase of *Pseudomonas aeruginosa* Inhibits Angiogenesis by Selective Cytotoxicity to Endothelial Cells

**DOI:** 10.1371/journal.ppat.1000420

**Published:** 2009-05-08

**Authors:** Michael L. Vasil, Martin J. Stonehouse, Adriana I. Vasil, Sandra J. Wadsworth, Howard Goldfine, Robert E. Bolcome, Joanne Chan

**Affiliations:** 1 Department of Microbiology, University of Colorado Denver, Anschutz Medical Center, Aurora, Colorado, United States of America; 2 Department of Microbiology, University of Pennsylvania School of Medicine, Philadelphia, Pennsylvania, United States of America; 3 The Vascular Biology Program, Children's Hospital Boston, and Department of Surgery, Harvard Medical School, Boston, Massachusetts, United States of America; Massachusetts General Hospital, United States of America

## Abstract

The hemolytic phospholipase C (PlcHR) expressed by *Pseudomonas aeruginosa* is the original member of a Phosphoesterase Superfamily, which includes phosphorylcholine-specific phospholipases C (PC-PLC) produced by frank and opportunistic pathogens. PlcHR, but not all its family members, is also a potent sphingomyelinase (SMase). Data presented herein indicate that picomolar (pM) concentrations of PlcHR are selectively lethal to endothelial cells (EC). An RGD motif of PlcHR contributes to this selectivity. Peptides containing an RGD motif (i.e., GRGDS), but not control peptides (i.e., GDGRS), block the effects of PlcHR on calcium signaling and cytotoxicity to EC. Moreover, RGD variants of PlcHR (e.g., RGE, KGD) are significantly reduced in their binding and toxicity, but retain the enzymatic activity of the wild type PlcHR. PlcHR also inhibits several EC-dependent in vitro assays (i.e., EC migration, EC invasion, and EC tubule formation), which represent key processes involved in angiogenesis (i.e., formation of new blood vessels from existing vasculature). Finally, the impact of PlcHR in an in vivo model of angiogenesis in transgenic zebrafish, and ones treated with an antisense morpholino to knock down a key blood cell regulator, were evaluated because in vitro assays cannot fully represent the complex processes of angiogenesis. As little as 2 ng/embryo of PlcHR was lethal to ∼50% of EGFP-labeled EC at 6 h after injection of embryos at 48 hpf (hours post-fertilization). An active site mutant of PlcHR (Thr178Ala) exhibited 120-fold reduced inhibitory activity in the EC invasion assay, and 20 ng/embryo elicited no detectable inhibitory activity in the zebrafish model. Taken together, these observations are pertinent to the distinctive vasculitis and poor wound healing associated with *P. aeruginosa* sepsis and suggest that the potent antiangiogenic properties of PlcHR are worthy of further investigation for the treatment of diseases where angiogenesis contributes pathological conditions (e.g., vascularization of tumors, diabetic retinopathy).

## Author Summary

*Pseudomonas aeruginosa* is a major bacterial opportunistic pathogen responsible for acute (e.g., sepsis) and chronic infections (e.g., pulmonary). While it expresses assorted extracellular toxins that in one way or another contribute to pathogenesis, the precise cellular and molecular mechanisms by which these factors act is largely unknown. During septicemia, *P. aeruginosa* frequently causes vasculitis and thrombosis. Endothelial cells line the entire vascular system of mammals and have sundry key functions in infectious diseases, including thrombosis and inflammation. Endothelial cells are essential to the formation of new blood vessels (angiogenesis) needed for proper wound healing. This report describes in vitro and in vivo experiments demonstrating that an extracellular toxin, PlcHR, at very low (picomolar) concentrations, is highly lethal to endothelial cells and inhibits angiogenesis in vivo. Data herein also suggest that PlcHR is selectively toxic to endothelial cells through its ability to bind a cell receptor(s). These observations may be particularly relevant to the mechanisms by which *P. aeruginosa* causes vascular lesions and inhibits the healing of wounds during septic infections. Finally, the potent antiangiogenic attribute of PlcHR could be useful in the treatment of noninfectious diseases where angiogenesis contributes their pathogenesis, including the vascularization of tumors and the eye (e.g., diabetic retinopathy).

## Introduction

The diverse roles that phospholipase C (PLCs) and sphingomyelinase (SMase) play in biology and medicine are extraordinary. Both types of phosphodiesterases and their substrate products (e.g. ceramide, diacylglycerol) have proven to be far more multifaceted than initially perceived, and their impact on a wide range of basic cellular processes in eukaryotes, including oncogenesis, apoptosis, and inflammation has been increasingly appreciated [1]–[3]. For example, Teichgräber et al. [4] recently reported that ceramide (CM) accumulation in the lungs of *Cftr*-deficient mice, resulting from the activation of an endogenous acidic SMase (ASMase), mediated a harsh inflammatory response. This led to wide spread apoptosis in pulmonary epithelial cells and an increased susceptibility to severe *Pseudomonas aeruginosa* infections. More recently, it was reported that a defective ASMase pathway in cystic fibrosis (CF) is perhaps a key contributor to the unabated IL-8 response during *P. aeruginosa* infections and to the failure of compromised hosts to eradicate bacterial colonization [5].

Likewise, there are sundry noteworthy functions for prokaryotic PLCs and SMases [6],[7]. One major class of bacterial PLCs is the Zn-dependent enzymes, which include α-toxin of *Clostridium perfringens* and PlcB of *Listeria monocytogenes*. Zn-dependent refers to the three Zn^2+^ atoms present in their active sites that are required for catalytic activity. Although α-toxin and PlcB both belong to this class of PLCs, their roles in pathogenesis are quite distinct. The α-toxin is markedly cytotoxic to eukaryotic cells and contributes to the severe myonecrosis associated with gas gangrene. By contrast, PlcB is important for the escape of this facultative intracellular pathogen from phagocytic vacuoles into the cytoplasm of macrophages, and other cell types infected with *L. monocytogenes* [8].

Some of the above bacterial phosphodiesterases act on phosphatidylcholine (PC), as well as sphingomyelin (SM). The α-toxin of *C. perfringens* and PlcB or *L. monocytogenes* hydrolyse either PC or SM to generate DAG or CM and phosphorylcholine, while a *Bacillus cereus* PC-PLC from the same zinc-dependent class of enzymes, has no activity whatsoever on SM [9]. The SMase activity of α-toxin however, is more critical to its role in pathogenesis than its PC-PLC activity [10]–[12].

A second major class of prokaryotic phosphorylcholine preferring PLCs, some of which are also SMases, is part of a large Phosphoesterase/PLC Superfamily [13]. These include bacterial and fungal PLCs, in addition to prokaryotic and eukaryotic phosphatases. The PLC members of this family are not Zn-dependent enzymes; rather divalent metals including Zn and nickel readily inhibit their activity [13]. For this and other reasons, Zn-dependent and Zn-independent bacterial PLCs each use distinct catalytic mechanisms to hydrolyze the phosphodiester bond between the phosphoryl head group of a choline containing phospholipid or sphingolipid, and their DAG or CM moieties [14].

The hemolytic phospholipase C (PlcH) of *Pseudomonas aeruginosa*, the focus of the present report, is the founding member of the large Zn-independent PLC family. Frank bacterial pathogens (i.e. *Mycobacterium tuberculosis*, *Bordetella pertussis* & *Francisella tularensis*), opportunistic bacteria (*P. aeruginosa*, *Acinetobacter baumannii*), fungal pathogens (*Aspergillus fumigatus*), as well as the Class B Select Agents *Burkholderia pseudomallei* & *mallei*, express homologous proteins belonging to the phosphodiesterase (i.e. PLC) part of the family [13]. Some pathogens (e.g. *Mycobacterium tuberculosis*, *B. pseudomallei*) carry three, or as many as four genes, encoding enzymes belonging to this class of PLCs [15]–[18]. All *P. aeruginosa* strains thus far sequenced (7) have two genes encoding members of this family, PlcH and PlcN [13]. In one way or another, many of these PLCs have now been associated with the virulence of the particular organism that produces them, but their contributions at the cellular level remain obscure.

PlcH, which is both a PC-PLC and a SMase, is a significant virulence determinant of *P. aeruginosa* in an array of infection models. Ostroff et al. showed that a PlcH deletion mutant had a 10,000-fold increase in its LD_50_ in a mouse thermal injury model, compared to that of its wild type parent [19]. Rahme et al. [20] confirmed the attenuated phenotype of a PlcH mutant in the same type of mouse infection model, but their mutant was constructed using a different parental *P. aeruginosa* strain (i.e. PA-14). They also reported that this PlcH mutant was attenuated in a plant (i.e. *Arabidopsis thaliana*) infection model. PlcH can be a noteworthy virulence factor in other non-mammalian hosts, as well. Hogan and Kolter reported that a PlcH mutant was attenuated in a *P. aeruginosa* infection of the mycelial phase, but not the yeast phase, of *Candida albicans* [21]. This particular mutant was one of the more attenuated ones in an assortment of other types of *P. aeruginosa* mutants they tested in this model.

Early on, PlcH was predominantly characterized for its hemolytic activity and enzymatic properties [13],[22]. More recent studies suggest that the SMase activity of PlcHR is much more critical than its PC-PLC activity, with regard to its ability to cause a phenomena known as “hot-cold hemolysis” as well as hemolysis at 37°C [13],[22],[23]. The present study however, began as an effort to assess its potential toxicity to a variety of nucleated mammalian (i.e. non-erythrocytic) cell lines, with the objective of discerning additional clues about its possible role at the cellular level in *P. aeruginosa* infections. In the present study, we report that PlcHR is a potent bacterial toxin, with selectivity for endothelial cells (EC) that is, at least in part, mediated through its Arg-Gly-Asp (RGD) motif. Based on its selective cytotoxicity for EC, PlcHR is a potent antiangiogenic agent in assorted assays (e.g. EC invasion assays, tube formation) that evaluate different stages of angiogenesis in vitro, and PlcHR inhibits angiogenesis in an in vivo model in zebrafish. These and other data, implicate PlcH in the pathogenesis of “vasculitis”, and the poor, angiogenesis-dependent, wound healing, typically observed in sepsis associated with this opportunistic pathogen [24],[25]. From a different perspective, it is possible that potent antiangiogenic bacterial toxins, such as Anthrax toxin and PlcHR, might also provide novel ways to mitigate the pathological consequences of abnormal angiogenesis (e.g. tumor vascularization, diabetic retinopathies).

## Results

### PlcHR is selectively cytotoxic to endothelial cells

**Figure 1:**
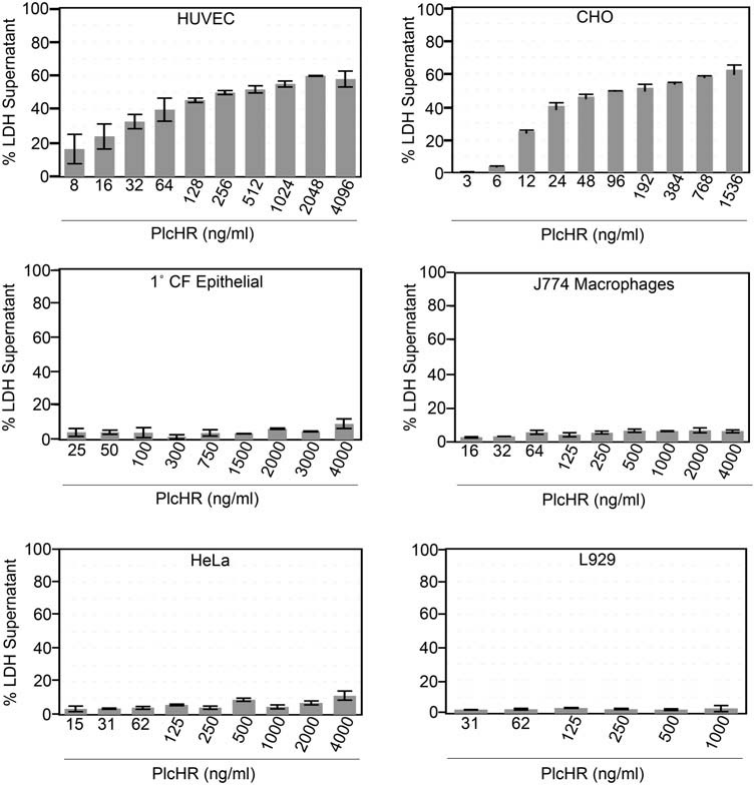
Cytotoxicity of PlcHR to assorted eukaryotic cells. Cytotoxicity (measured by LDH release) for assorted eukaryotic cell cultures using the CytoTox 96 Assay Kit as described in Materials and Methods. Concentrations of PlcHR (ng/ml) used are noted, and LDH release was measured after 6 h of incubation.

PlcHR, a complex heterodimer, consisting of PlcH and PlcR, was expressed in *P. aeruginosa* and purified as previously described [13],[22],[23]. PlcHR is hemolytic to human and sheep erythrocytes, and had been previously reported to be moderately cytotoxic to neutrophils and macrophage [22],[23],[26],[27]. Yet, no one had conducted a systematic evaluation of its cytotoxic potential for other types of nucleated cells. Accordingly, the cytotoxic effects of PlcHR toward an assortment of other types of eukaryotic cells was undertaken using the following cell lines: a 1° human lung cell line from a CF patient [28]; HeLa (epithelial); L929 mouse fibroblasts; J774 macrophage; Chinese hamster ovary cells (CHO); and Human Umbilical Vascular Endothelial Cells (HUVEC). As shown in Figure 1 and Figure S1, remarkably low concentrations (<10 ng/ml) of PlcHR were distinctly cytotoxic to HUVEC and CHO cells. By contrast, much higher concentrations of PlcHR (i.e. >4 µg/ml) were only slightly cytotoxic to any of the other cell types examined including, 1° CF lung epithelial cells and J774 macrophage, that were evaluated at 6 hrs after the application of varying amounts of PlcHR. An additional epithelial cell line, A549 lung cells was also tested, but they were even more resistant to the cytotoxic effects of PlcHR than the 1° CF lung epithelial cells were (data not shown).

**Figure 2:**
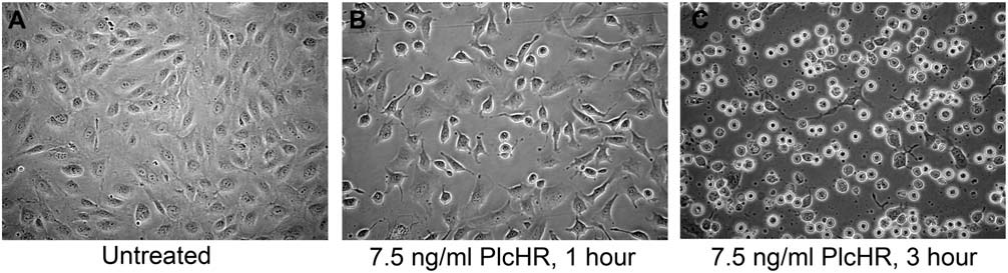
Treatment with pM (PlcHR MW = 95.6 kDA) concentrations of PlcHR induces morphological changes, but not direct lysis of EC. (A) Untreated HUVEC. (B) HUVEC treated with 7.5 ng/ml PlcHR_2_ for 1 h. (C) HUVEC treated with 7.5 ng/ml PlcHR_2_ for 3 h. Pictures are at 200× magnification.

As shown in Figure 2, PlcHR does not seem to cause lysis of EC even after 1 hour (Figure 2B), as would be expected if it had been simply hydrolyzing their membranes through its PLC/SMase activity. Moreover, because the outer leaflets of the membranes of all of these cells contain equimolar concentrations of PC and SM [29], these data suggest that the enzymatic activities of PlcHR, per se, are not sufficient to account for its EC and CHO cell selectivity, compared to the other types of cells that were tested (Figure 1 and Figure S1).

### PlcHR induces a rise in intracellular calcium in EC

An association between calcium and the hemolytic activity of PlcHR has been previously noted [13], and associations between the calcium responses of eukaryotic cells to other bacterial PLCs have been reported [7]. Based on those observations, the effect of PlcHR on intracellular calcium levels in EC was investigated. Only 5 ng/ml of PlcHR induced a significant increase in intracellular calcium levels in EC, which peaked at 7 minutes (Table 1). The rise in calcium levels could originate from the influx of extracellular calcium or via the release of calcium from stores in the endoplasmic reticulum (ER). The two possible pathways by which intracellular calcium increases can be separated by pretreatment of EC with the calcium channel blocker SK&F96365 for 15 min (inhibits entry of extracellular calcium via calcium channels) or by pretreatment with thapsigargin for 45 min (depletes intracellular calcium stores in the endoplasmic reticulum [30],[31]). The 45 minute pretreatment of EC with thapsigargin (1 µM), before application of 5 ng/ml of PlcHR (Table 1), significantly blocked the subsequent rise in intracellular calcium levels, that is normally observed in EC treated only with PlcHR (5 ng/ml) (Table 1). Intracellular calcium levels at 7 minutes in EC treated with thapsigargin and PlcHR was 55±6 nM, while intracellular calcium levels at 7 minutes in EC treated only with PlcHR was 568±58 nM. In contrast, there were no significant differences in the intracellular calcium levels between cells that were pre-treated with 25 µM of SK&F96365, and then subsequently treated with PlcHR (435±43 nM), compared to those treated only with PlcHR (429±48 nM) (Table 1). When PlcH alone was compared to PlcHR a more rapid (>2 fold) release of intracellular calcium in EC was observed, but their maximums were ultimately virtually the same (data not shown). Also, >100 ng/ml of PlcHR failed to induce any increase in intracellular levels of calcium in J774 macrophage (data not shown), which are resistant to the cytotoxicity of PlcHR (Figure 1).

**Table 1:** PlcHR calcium signaling and mechanism of intracellular calcium release.

Time (min)	Untreated EC nM Calcium	Treated EC (5 ng/ml PIcHR) nM Calcium
1	56±2.3	129±25
3	50±1.2	306±47
5	32±10.5	362±44
7	31±5.6	568±58**
10	99±12	435±48*
12		357±23
15		229±31
17		189±68
20	72±15	190±25
25		115±18
30		66±9.8

*: -p≤0.01 for HUVECS+PlcHR at 7 and 10 min compared to untreated cells.

**: Thapsigargin (TG) results (EC cells pretreated with TG 45 min prior PlcHR treatment): 7 min untreated EC (no PlcHR), 62±6.8 nM; 7 min EC+PlcHR no TG, 515±72 nM**; 7 min EC+PlcHR+TG, 55±6 nM; 7 min EC no PlcHR+TG, 76±13.5 nM. SK&F96365 (SK) results (EC cells pretreated with SK 15 min prior PlcHR treatment): 15 min untreated (no PlcHR), 62±6.8 nM; 15 min EC+PlcHR no SK, 429±48 nM; 15 min EC+PlcHR+SK, 435±43 nM.

These data indicate that the types of cells that are susceptible or resistant to calcium signaling by PlcHR reflect the type of cells that are susceptible or resistant to its cytotoxicity. Because PlcHR induces the release of calcium from the ER in EC, rather than through calcium channels, it is particularly relevant to note that calcium release from the ER, will in turn, activate downstream effectors of apoptosis (e.g. calcineurin, calpains, endonucleases) [32]. Finally, with regard to the PC-PLC and SMase activity of PlcHR, it is important to note that increased levels of ceramide (a SMase product), but not diacylglycerol (a PC-PLC product), can induce the release of calcium from ER stores [33].

### Induction of caspase-3 activity by PlcHR

**Figure 3:**
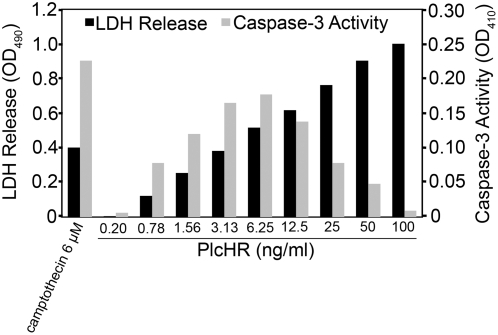
Caspase-3 activation in EC by PlcHR. After 12 h of the treatment of EC with varying amounts (ng/ml) of PlcHR (gray bars), measurement of caspase-3 activation was performed as described in Materials and Methods. Camptothecin was used as a positive control of caspase-3 activation. Measurement of LDH release (black bars) was performed as described in Materials and Methods.

The aspartate-specific cysteinyl proteases or caspases are a set of mediators implicated in apoptosis. The activation of caspase-3 in mammalian cells is a hallmark of apoptosis [34]. While the generation of DAG from PC would be expected to induce EC proliferation, hydrolysis of membrane SM by PlcHR, would cause a relatively increase in CM, leading to programmed cell death (i.e. apoptosis) [33]. We therefore chose to examine whether PlcHR cytotoxicity could induce increased levels of caspase-3 activity, which is one of the effector caspases that ultimately carry out apoptosis. As shown in Figures 3, S2A and S2B, treatment of EC with pM concentrations of PlcHR resulted in activation of caspase-3. In experiments shown in Figure 3, both caspase-3 activation and LDH release were measured at 16 hours post treatment with increasing concentrations of PlcHR. Figure 3 shows that caspase-3 activity increases as the concentration of PlcHR increases until it peaks at 6.25 ng/ml PlcHR. The level of caspase-3 activity induced with 6.25 ng/ml PlcHR is similar to cells treated with the apoptosis control compound camptothecin (Figure 3). Beyond 6.25 ng/ml PlcHR, caspase-3 activity begins to decrease, but the release of LDH continues to increase until ultimately at 100 ng/ml PlcHR there is very little caspase-3 activity and LDH release has reached its maximum. The addition of the pan-caspase inhibitor Z-VAD-FMK completely inhibited PlcHR activation of caspase-3 and reduced the level of LDH release (Figure S2B), indicating that a significant portion of the cells releasing LDH are actually dying by apoptosis

Finally, a time course for activation of caspase-3 was performed to determine when caspase-3 was activated by treatment with PlcHR. As shown is Figure S2A treatment of EC with as little as 2.5 ng/ml of PlcHR activated caspase-3 between 3 and 6 hours. Caspase-3 activation was never detected in resistant cells (e.g. Hela) when they were treated with >100 ng/ml of PlcHR (data not shown).

### Selective toxicity of PlcHR is associated with its RGD motif

PlcH has an RGD motif (Figure S3), which is present in only one other PLC (i.e. one of the three PlcH homologs expressed by *Burkholderia thailandensis*) of the now >50 other members of this class of enzymes. RGD motifs are associated with cell-cell and cell-matrix interactions and they play a major role in host cell recognition by assorted viruses, as well as bacterial virulence factors (e.g. invasin, toxins) through their interactions with cell surface integrin receptors [35]. Yet, the context of an RGD motif in a protein is likewise critical to its ability to bind integrins [36], and variants of this motif (i.e. RGE, KGE) may still be functional for binding integrin receptors [37],[38].

Several approaches were taken to assess whether the RGD motif of PlcH is associated with its selectivity for EC or CHO cells. PlcHR and free PlcH caused a significant increase in the level of intracellular calcium release that peaked at 7 minutes (Tables 1 and 2). However, when a 5-mer peptide with an RGD motif (i.e. GRGDS) was added at the same time as PlcHR, or free PlcH, there was no evidence of release of calcium from ER stores over that seen in untreated cells. By contrast, when a 5-mer peptide with a scrambled RGD sequence (i.e. GDGRS) was added to EC in conjunction with PlcH, an increase in the intracellular calcium release reaching a maximum at 7 min, comparable to that seen with PlcHR alone, was observed (Tables 1 and 2).

**Table 2:** RGD peptide blocks PlcHR-induced increase in intracellular calcium.

Sample	Maximum
PlcHR only	551±91
PlcHR+RGD peptide	127±31
FreePlcH only	699±67
Free PlcH+RGD peptide	129±48
RGD peptide only	120±26
DGR peptide only	105±19
Free PlcH+DGR peptide	634±50

EC were incubated with either GRGDS or GDGRS at a final concentration of 191 nM for 30 min before the addition of PlcHR_2_ or free PlcH at a final concentration of 191 pM.

The ability of these peptides to attenuate the cytoxicity of PlcHR was also evaluated. Similar effects of these peptides were seen (Table 3). The RGD containing peptide (GRGDS) at approximately a 50-fold molar excess of peptide to PlcHR significantly dampened the lethal impact of PlcHR, while the scrambled peptide (GDGRS) at the same concentration demonstrated no significant effect on PlcHR toxicity in this assay.

**Table 3:** RGD peptide blocks PlcHR-mediated cytotoxicity.

Sample	n	Mean	SEM	95% Confidence Interval	T	P
PlcHR_2_ (45 pM)	9	46.5	1.27	44% to 49%	—	
PlcHR_2_ (45 pM) G**RGD**S 2.4 µM	6	35.6	0.9	33% to 38%	5.91	0.000051
PlcHR_2_ (45 pM) G**DGR**S 2.4 µM	3	45.7	0.91	41% to 50%	0.742	0.339

Cells were pre-incubated with the peptides for 15 min prior to the addition of PlcHR and were maintained during the 6 h incubation with PlcHR_2_. Cytotoxicity was measured via LDH release assay. G**RGD**S peptide and control G**DGR**S peptide alone had no observed effect on the cell viability.

**Figure 4:**
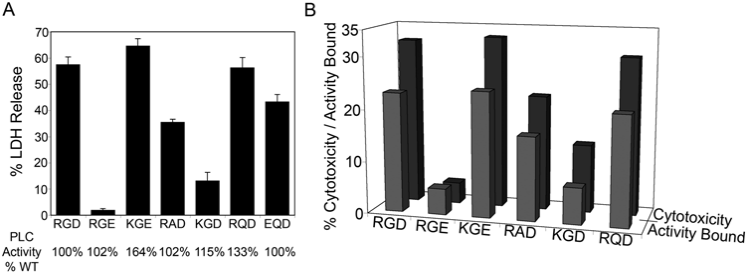
Evaluation of the binding and cytotoxicity of wild type PlcHR and RGD mutants to susceptible cells. (A) Cytotoxicity (LDH release) of wild type PlcHR and six RGD mutants to EC. (B) Analysis of binding (see protocol in Materials and Methods) and cytotoxicity of PlcHR wild type and five RGD mutants to CHO cells. The PLC activities of wild type PlcHR and the RGD variants were evaluated before application to the EC and CHO cells, and cytotoxicity (EC & CHO) and binding (CHO) of each mutant is presented as percent of the wild type PlcHR.

Additionally, several RGD variants of PlcHR were constructed. Various *plcH* genes encoding PlcH with altered RGD motifs were expressed, along with the wild type *plcR* gene, and the resulting PlcHR-RGD mutant proteins were purified. Each contain variant RGD motifs (e.g. RGE, RAD, RQD) that are found in other PlcH homologs in the NCBI database (Figure 4). Several RGD variants were then evaluated for their enzymatic activity (Figure 4A), as well as, their binding (Figure 4B) and cytotoxicity to EC or CHO cells (Figure 4A & 4B). In support of the hypothesis that the RGD motif of PlcHR is a critical factor in its ability to kill EC or CHO cells, some variants (i.e. RGE, KGE), still retained wild type PLC activity, but were significantly decreased in their binding or lethality to EC or CHO cells by comparison to the wild type PlcHR (Figure 4A & 4B). Finally, we found that a resistant cell line (i.e. L929 fibroblasts) bound significantly less toxin (16% of total PlcHR added) compared to EC (42% of total PlcHR added) and that binding of PlcHR to EC was saturable. That is, there was no increased binding despite higher concentrations of PlcHR added to these cells (Stonehouse, M. doctoral thesis University of Colorado Denver).

### PlcHR inhibits angiogenesis in vitro

Based on the selective and potent toxicity of PlcHR toward EC, it was of interest, particularly in the context of vasular disease associated with *P. aeruginosa* sepsis (see Discussion below), to examine whether this EC selective toxin inhibits the more complex processes associated with angiogenesis (i.e. formation of new vessels from the existing vasculature). There are assorted in vitro angiogenesis assays (e.g. migration and invasion assays, tube formation) to approximate individual mechanisms involved in the formation of new blood vessels. See reference [39] for a detailed description of the angiogenesis assays described below.

**Figure 5:**
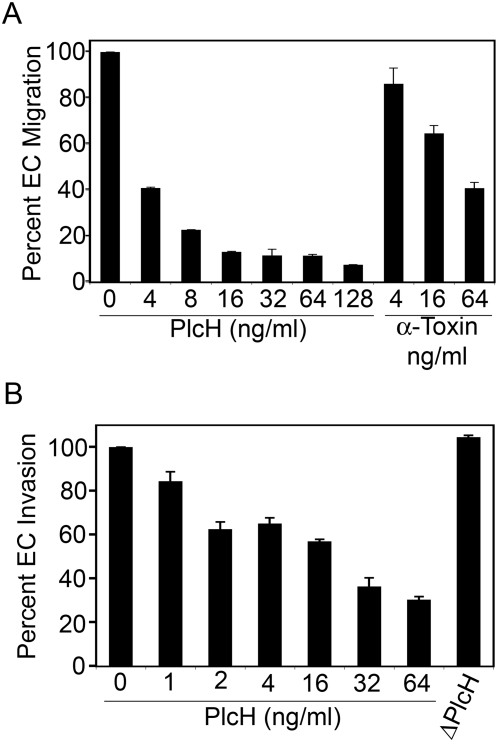
In vitro angiogenesis assays. Effect of PlcHR on EC migration (A) and EC cell invasion (B) in the presence of varying concentrations of PlcHR shown in these figures. Assays were performed as described in Materials and Methods. In both assays vascular endothelial growth factor (VEGF) was used as the chemoattractant. The α-toxin of *Clostridium perfringens* used in the migration assays (A) was generously provided by Graeme Clarke and Richard Titball. 100 ng/ml of heat-inactivated (95°C, 10 min) PlcHR (ΔPlcHR) was evaluated for inhibition in the (B) EC invasion assay.

The EC migration assay is based on the movement of cells through a fluorescence blocking, microporous inert filter coated with human fibronectin. The pores of the membrane are not occluded, which allows the EC to attach to the membrane and freely migrate toward the angiogenic stimulus, vascular endothelial growth factor (VEGF), in a chamber below. A fluorescent plate reader is used to quantify EC, labeled with a fluorescent dye, which migrated through the filter. In the EC migration assay (Figure 5A) only 3 ng/ml of PlcHR was required to inhibit EC migration by 50%, (IC_50_) while the IC_50_ for an unrelated PLC/SMase, *C. perfringens* α-toxin (generously provided by Graeme Clarke and Richard Titball) was 45 ng/ml (Figure 5A).

The EC invasion assay is performed in a similar manner however, the filter in this assay is coated with a Matrigel Matrix, which is a biologically active basement membrane preparation derived from the Engelbreth-Holm-Swarm mouse tumor. This coating blocks the passage of non-invasive cells while allowing the passage of activated EC. Because the membrane blocks any fluorescence coming from the upper surface of the membrane, only fluorescence from cells that have invaded through the basement membrane are detected. A fluorescence plate reader is used to quantify the labeled cells, as with the migration assay. With the EC invasion (Figure 5B) assay the IC_50_ of native PlcHR was 10 ng/ml, but >100 ng/ml of a heated PlcHR preparation (10 min @ 95°C) showed no inhibition in the EC invasion assay (Figure 5B).

**Figure 6:**
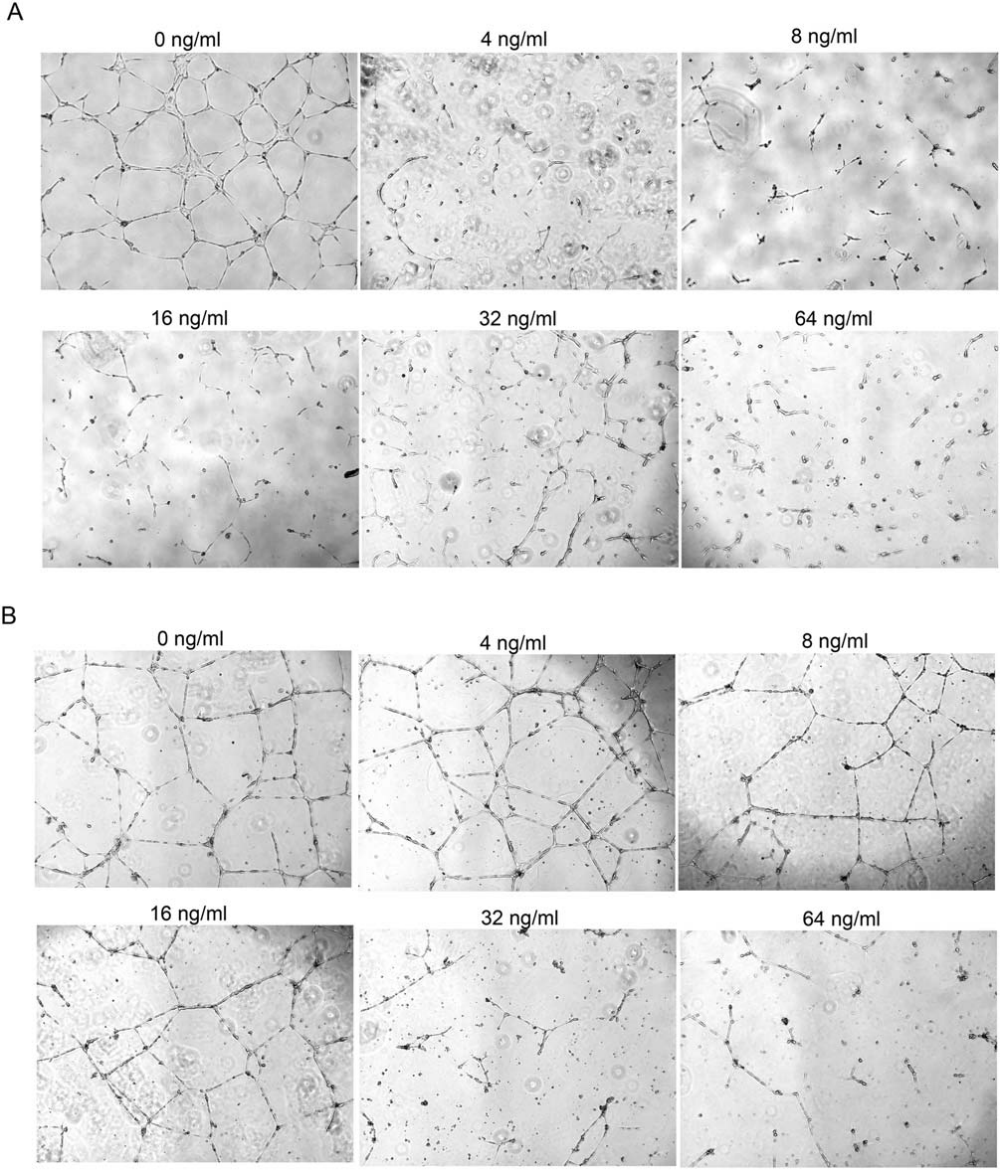
PlcHR inhibits tube formation by EC. (A) EC were challenged with 4–64 ng/ml of PlcHR during tube formation. Tube formation was then measured at 48 h on matrigel matrix. (B) EC that had already formed tubes after 24 h were then subsequently challenged with 4–64 ng/ml of PlcHR for an additional 24 h. Photographs were taken at 40× maginification.

The in vitro tube, or tubule, assay is regarded as one that represents the later stages of angiogenesis and is considered to be a model for in vivo capillary development. This method has been extensively employed to evaluate novel compounds for their antiangiogenic properties. In this assay EC differentiate into capillary-like structures (i.e. tubes), which contain a lumen surface surrounded by cells, which display cell membranes that are connected to one another by junctional complexes, indicative of in vivo-capillary formation. Only 4 ng/ml of PlcHR completely disrupted tube formation when it was applied during the formation of the tubes (Figure 6A and Table S1). However, if tubes were allowed to form before PlcHR was applied, it took ∼8-fold higher concentrations of PlcHR (i.e. 32 ng/ml) to completely disrupt tube formation (Figure 6B and Table S1). In both cases, 64 ng/ml of a heated preparation of PlcHR had no effect on tube formation (data not shown). It should be noted that when the media containing PlcHR2 was exchanged with fresh media after the initial 24 hour incubation, tubes formed within 24 hours indicating that PlcHR2 was not necessarily killing the endothelial cells but was inhibiting the tube formation process (data not shown). These and other data (Stonehouse and Vasil unpublished observations) suggest that proliferating EC might be more sensitive to PlcHR than EC already established in a vascular structure.

### Characterization of a PlcH-active site mutant

**Figure 7:**
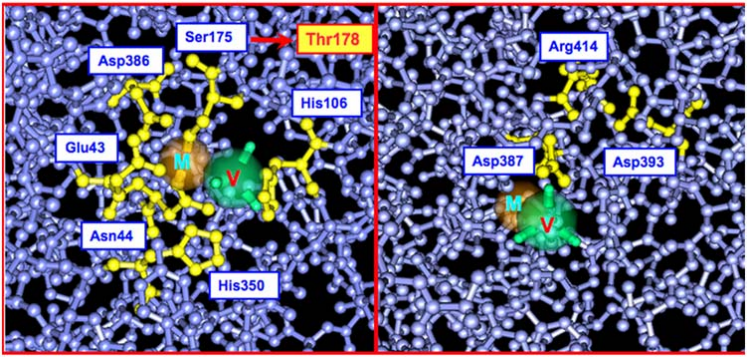
Alignment of active site residues of *Francisella tularensis* AcpA with PlcH. The amino two-thirds of AcpA and PlcH share 23% identity. Five of the ten AcpA active site residues are identically conserved in PlcH and AcpA. The Thr178 (Ala in the PlcH Thr178Ala mutant) aligns with the AcpA nucleophile Ser-175 (left side). The Glu-358 in PlcH aligns with an AcpA metal, possibly calcium (orange ball marked M, both frames) binding residue Asp-387, and ion pair residues Asp-393 and Arg-414 of AcpA are also found (Asp-364, Arg-401) in the PlcH sequence (right side). A green ball marked V in both right and left frames denotes the AcpA and PlcH competitive inhibitor, phosphate analog, vanadate.

We sought to provide evidence that the cytotoxicity of PlcHR is dependent on its enzymatic activity. Although presently, there is no crystal structure data for PlcHR, there is a member of the Phosphoesterase/PLC Superfamily of proteins, which, based on sequence similarities to other members, is situated just at the evolutionary border between the known PLC members, and those, which are only phosphatases [13],[14]. This protein, AcpA (with 23% identity to two thirds of the amino terminus of PlcH), is a significant virulence factor of *Francisella tularensis*. AcpA appears to play a role in his pathogen's intracellular trafficking in macrophages [40]. AcpA is a periplasmic protein with acid phosphatase activity that can efficiently use an assortment of biologically significant phosphorylated compounds as substrates (e.g. tyrosine-PO_4_ and ATP) [41]. Although AcpA is able to cleave the synthetic PLC substrate, ρ-nitrophenyl-phosphorylcholine (NPPC), in which a nitrophenyl group replaces the diacylglycerol (DAG) moiety of phospholipids (i.e. PC), AcpA cannot cleave the phosphodiester bond between the head group and the DAG or SM moiety of authentic phospholipids or sphingolipids (e.g. PC or SM) [14],[41]. Despite its questionable role as a legitimate PLC, analysis of the structure of AcpA has provided valuable insight into the location of the active site of PlcH [14] especially since it is the only member of the entire Phosphaesterase/PLC superfamily for which a crystal structure is available. The molecular architecture of AcpA was determined through an analysis of crystals formed in the presence of the competitive phosphatase inhibitor orthovanadate. Vanadate is also an inhibitor of PlcH activity (Vasil, M. and Stonehouse, M. unpublished observations) and it is an analog of the phosphate ion that would be present in an AcpA substrate (e.g. tyrosine-PO_4_) or as a phosphate that links the head group (e.g. choline) of a phospholipid or sphingolipid (PC or SM) to diacylglycerol or ceramide. Because a phosphatase and a PLC both cleave a phosphoester bond, specifically a phosphodiester bond in the case of PlcH, the arrangement of the orthovanadate in the AcpA structure provided some significant information about the amino acids in the active site of AcpA, as well as PlcH. Five of the ten AcpA active site residues are identically conserved in PlcH (Figure 7). Additionally, Thr-178 of PlcH aligns with the nucleophile Ser-175 of AcpA (Figure 7). Prior to the publication of the AcpA structural data, a Thr178Ala mutant of PlcH was constructed, expressed and purified by the same methods used for native PlcHR (see Materials and Methods). The enzyme kinetics of the mutant and the wild type PlcHR were evaluated using NPPC as the substrate [13] providing the following results: PlcHR wild type; V_max_(µmol·min^−1^·mg^−1^) = 147±6; K_m_ (µM) = 19±2.9; **k_cat_ (s^−1^) = 192**: PlcHR Thr178Ala; V_max_ (µmol·min^−1^·mg^−1^) = 4.85±1; K_m_ (µM) = 200±57; **k_cat_ (s^−1^) = 6**. The structural AcpA data, along with the enzymology data above, suggest that Thr178 is an active site residue in PlcH.

With regard to its biological activity, >500 ng/ml of the Thr178Ala mutant showed less than 5% cytotoxicity to EC in the LDH release assay and its IC_50_ in the EC invasion assay was 1200 ng, as compared to an IC_50_ of 10 ng/ml for the wild type PlcHR (data not shown). Finally, the in vivo antiangiogenic activity of the Thr-178-Ala mutant was severely attenuated in the zebrafish model and 20 ng of the Thr178Ala mutant failed to cause any of the phenotypes observed in this model with only 2 ng of wild type PlcHR (Table 4).

**Table 4:** Dose responses in zebrafish embryos to PlcHR.

Dose (ng)	HPI	Vessel Collapse	No Circulation	Pericardial Edema	Altered Heart Morphology
1 PlcHR	1	88%	4%	—	—
1 PlcHR	6	75%	4%	—	—
1 PlcHR	12	50%	4%	—	—
1 PlcHR	24	—	—	—	—
2 PlcHR	1	100%	100%	—	—
2 PlcHR	6	100%	100%	100%	100%
2 PlcHR	12	100%	100%	100%	100%
2 PlcHR	24	100%	100%	100%	100%
10 PlcHR-T178A	1	—	—	—	—
10 PlcHR-T178A	6	—	—	—	—
10 PlcHR-T178A	12	—	—	—	—
10 PlcHR-T178A	24	—	—	—	—

Embryos injected at 48 hpf. 2 ng PlcHR, n = 28; 1 ng PlcHR, n = 24; 10 ng PlcHR-T178A, n = 20.

### PlcHR targets and kills EC in the zebrafish model

**Figure 8:**
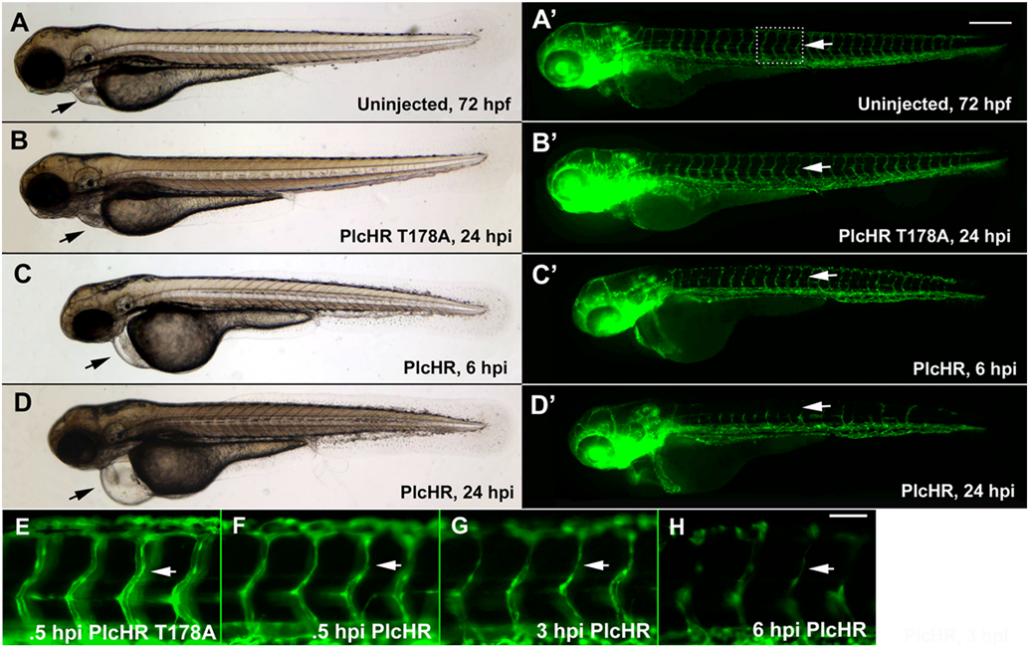
PlcHR causes cardiovascular effects in the zebrafish. Transgenic zebrafish embryos expressing EGFP in endothelial cells, (Tg(Fli:EGFP) [41]) were utilized to examine PlcHR effects. (A–H) Embryos at 48 hpf (hours post-fertilization) were injected with 2 ng of PlcHR, or a catalytically inactive mutant PlcHR T178A, and examined over the next 24 h. PlcHR T178A-injected embryos appeared phenotypically similar to controls (A,B). Prime panels denote visualization of EGFP-labeled endothelial cells in the same embryo. (C) At 6 hpi (hours post-injection), embryos injected with PlcHR had little or no circulation and pericardial edema (black arrows). (D) Effects became more pronounced by 24 hpi as intersegmental vessels regress (ISV, white arrows). (E–H) Higher magnification of the ISVs revealed that their lumens collapsed before endothelial cell regression. Representative pictures of embryos from one of three independent experiments; n>20 per condition. Scale bars indicate 250 µm for (A–D), 50 µm for (E–H).

**Figure 9:**
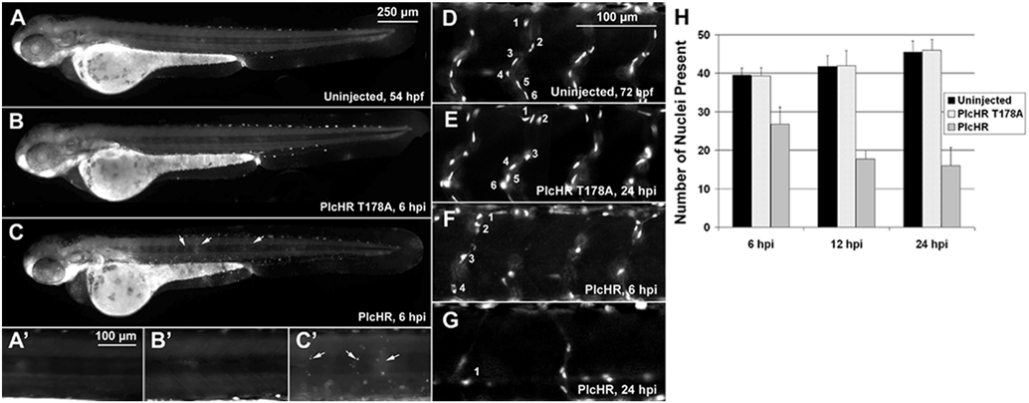
PlcHR reduces endothelial cell numbers in zebrafish embryos. (A–C) Acridine orange staining was used to indicate cell death at the location of endothelial cells in wildtype embryos injected with 2 ng of PlcHR at 6 hpi (arrows). Corresponding prime panels are enlarged areas showing cell death. (D–H) Zebrafish embryos expressing endothelial nuclear EGFP (Tg(Fli:nucEGFP) [46]) were utilized to assess PlcHR effects on endothelial cell numbers. As before, embryos at 48 hpf were injected with 2 ng of PlcHR, or PlcHR T178A, and compared with uninjected controls. To standardize the region used for counting nuclei, only those in the 8 ISVs immediately anterior to the cloaca were recorded [42]. (D,E,H) At 6, 12, and 24 hpi, there was no significant difference between PlcHR T178A-injected and uninjected animals. (C–F) Those injected with PlcHR showed a clear reduction in endothelial cell numbers by 6 hpi (*P*<.001). (H) Data taken from three independent experiments; n = 6, statistics performed by t-test.

**Figure 10:**
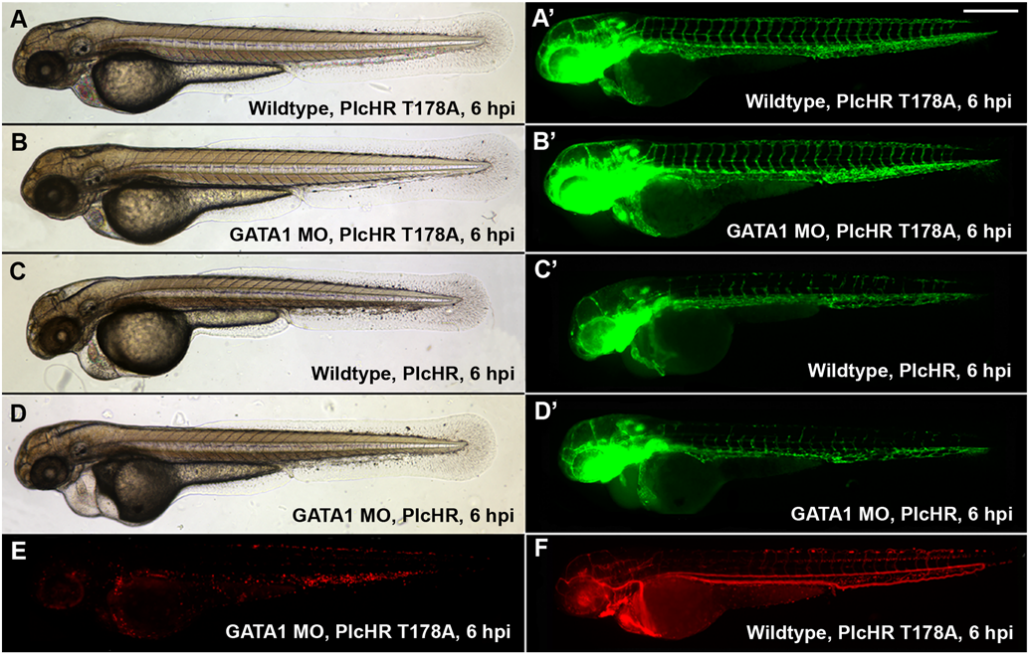
PlcHR acts on endothelial cells independently of erythrocytes wildtype (*Tg(Fli:EGFP)*) [44], and GATA1 morphants were injected with PlcHR or PlcHR T178A at 48 hpf and observed for phenotypes (n>20 per condition). PlcHR induced the same effects in morphants as were observed in wild type embryos (C,D), while no effects were noted in either group of embryos when injected with PlcHR T178A (A,B). Prime panels show effects on endothelial cells. (E,F) Depict the lack of circulating blood cells in GATA1 morphants and their presence in wildtype (*Tg(fli1:EGFP):Tg(gata1:dsRED)*) [44],[45] embryos at the time point in which embryos were scored, respectively. Scale bar indicates 250 µm.

**Figure 11:**
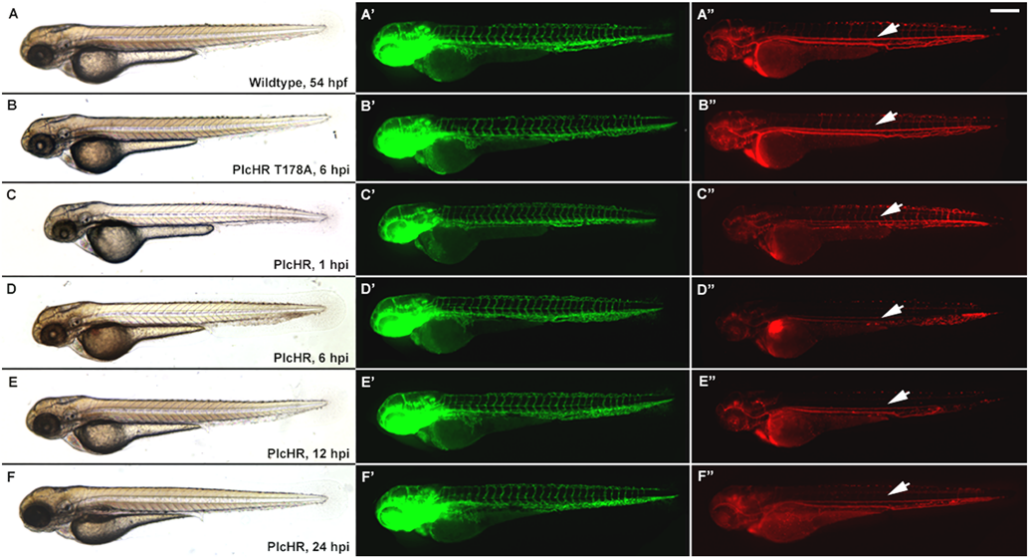
Zebrafish embryos display vascular collapse and then recover over a 24 h time period at low PlcHR doses. Transgenic zebrafish embryos expressing EGFP in endothelial cells and RFP in erythrocytes (*Tg(fli1:EGFP):Tg(gata1:dsRED)*) [41],[42]) were injected with 1 ng of PlcHR, or its catalytic mutant, as before. (A–F) Single and double prime panels denote visualization of EGFP-labeled endothelial cells or RFP-labeled blood cells in the same embryos, respectively. At 6 hpi, embryos injected with PlcHR displayed little or no circulation (compare D″ with A″, B″). (E–F) Circulation was partially restored by 12 hpi, and embryos recovered by 24 hpi. Representative pictures of embryos from one of three independent experiments; n>20 per condition. White arrow denotes blood flow in dorsal aorta. Scale bar indicates 250 µm.

**Figure 12:**
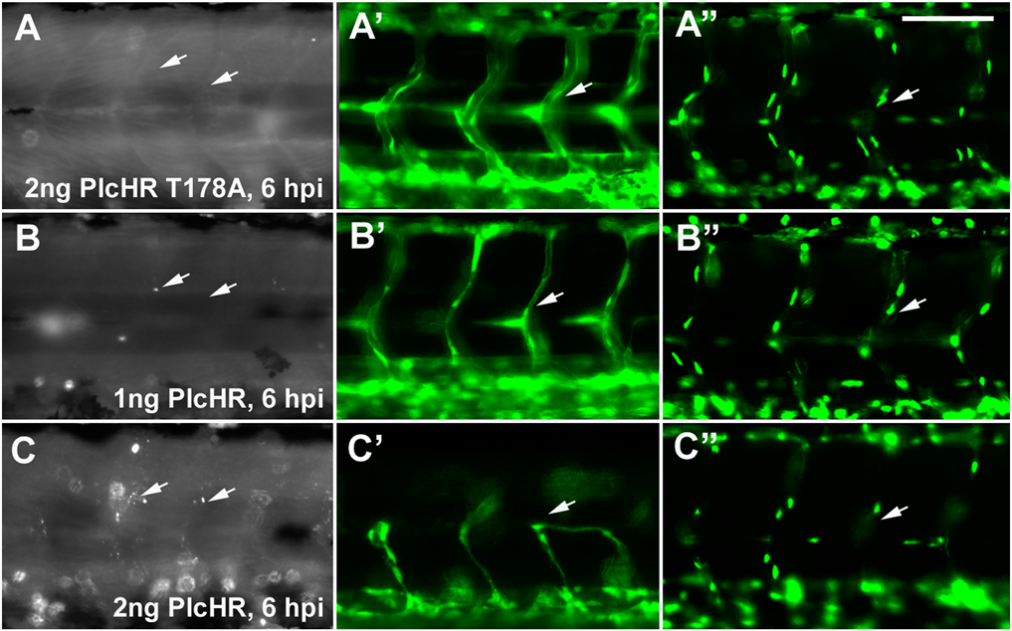
Endothelial cell death does not play a predominant role in zebrafish circulatory collapse and recovery following a 1 ng dose of PlcHR. (Tg(Fli:EGFP) [41]) and (Tg(Fli:nucEGFP) [Roman et al., 2002]) embryos were injected with 1 or 2 ng of PlcHR, or 2 ng of its catalytic mutant as before (A–C). (A–C) Acridine orange staining detected minimal cell death following the injection of 1 ng PlcHR (A′,B′), ISVs collapsed, but did not regress as in the 2 ng dose (C′), and the endothelial nuclei did not decrease [(A″,B″) and data not shown]. (C,C′,C″) The 2 ng PlcHR dose generated increased cell death, vessel regression, and reduced endothelial cell nuclei. Scale bar indicates 100 µm.

The zebrafish embryonic vasculature is highly accessible to the study of endothelial cell function, from the earliest differentiation of angioblasts (vasculogenesis) to the formation of new vessels from the existing vasculature (angiogenesis). Accordingly, zebrafish embryos were used to evaluate the effects of PlcHR on EC in vivo. PlcHR or the active site mutant, PlcHR-Thr178Ala, were injected directly into the circulation of embryos at 48 hours post fertilization (hpf) following an established protocol (Figure 8) [42]. Zebrafish embryos are transparent and have functional cardiovascular system by this time [43]. Transgenic zebrafish embryos expressing enhanced green fluorescent protein (EGFP) in endothelial cells, (Tg(fli1:EGFP) [44],[45] were utilized to observe PlcHR action on these cell types directly at the various times after the injection of these proteins (hpi – hours post injection). At 2 ng of PlcHR 6hpi, the embryos had significantly impaired circulation and pericardial edema (Figure 8C and C′) which, became more pronounced by 24 hpi (Figure 8D and D′) as intersegmental vessels (IVS, white arrows). Higher magnification of these IVS (Figures 8E–8H) revealed that their lumens collapsed before endothelial cell regression. Interestingly, PlcHR induced dosage-dependent defects were best observed at 1–2 ng (Table 4). At doses of 1 ng and lower, embryos exhibited decreased circulation and blood pooling in the venous plexus within 15 min following injection; however, they recovered by 24 hours post injection (hpi), to look comparable to wild type embryos (Table 4). At 2 ng of PlcHR, the same defects became progressively more severe until all embryos had no blood circulation, pericardial edema, and altered heart morphology, by 24 hpi (Table 4). At doses higher than 2 ng, the same defects occurred earlier and rapidly became more severe. At 10 ng PlcHR, necrosis developed in the heart, followed by the entire body within one hour. In contrast, no vascular or other phenotypic changes were observed in embryos injected with PlcHR-Thr178Ala, at doses up to 20 ng (Figures 8, 9, 10, 11 and 12). We used the 2 ng dose for subsequent experiments focusing on PlcHR's endothelial effects.

To examine PlcHR effects on endothelial cell death, we used a transgenic line with endothelial nuclear expression of EGFP *(Tg(fli:nucEGFP)^y7^* [46]). For this assay, endothelial cells within the 8 ISVs immediately anterior to the cloacae were counted [42]. At 2 ng PlcHR, we noted an ∼50% decrease in endothelial cell nuclei number in these vessels by 6 hpi. Nuclei counts continued to drop up to 24 hpi. Acridine orange staining was used to verify that the observed decrease in endothelial cell nuclei numbers was due to cell death (Figure 9).

PlcHR is also hemolytic to some, but not all mammalian and avian erythrocytes that have been examined. For example, PlcHR is hemolytic to Human, Sheep and Rabbit erythrocytes, but Horse, Ox and Cow erythrocytes are resistant to the hemolytic effects of PlcHR (Vasil, M and Vasil, A. unpublished observations). In our experiments we observed that blood cells ceased circulating and decreased in number over a 24 h time course at doses of 2 ng and higher (Figures 10, 11). To test whether PlcHR any of the effects on the cardiovascular system or endothelial cells were due to its action on blood cells, we used an antisense morpholino knockdown of GATA-1, an important blood cell regulator, using an established morpholino [47]. Morpholino injected embryos, called morphants, have dramatically reduced circulating red blood cells as compared with control, wild type embryos (Figures 10, 11). We injected either PlcHR or PlcHR-Thr178Ala and visually monitored embryos for effects at regular intervals over a 24 h period. We found that endothelial cells were still responding to PlcHR or PlcHR-Thr178Ala in a similar manner, in wildtype and morphant embryos (Figures 10, 11). Finally, we examined whether EC death plays a prominent role in circulatory collapse and recovery following a 1 ng dose of PlcHR. This was done by injecting (Tg(Fli:EGFP) and (Tg(Fli:nucEGFP) embryos [41] with 1 or 2 ng of PlcHR or the Thr178Ala catalytic mutant (Figure 12). Acridine orange staining detected minimal cell death following the injection of 1 ng of PlcHR, ISV's collapsed, but did not regress as in the 2 ng dose (Figure 12 C′) and the EC nuclei did not decrease (Figure 12 A″, B″). By contrast, the 2 ng dose of PlcHR generated increased cell death, vessel regression and reduced EC nuclei (Figure 12 panels C, C′ and C″). In all cases, 2 ng of the active site mutant of PlcHR, Thr178Ala, showed no effects.

## Discussion

Experimental data presented in this report have defined an EC-selective role for a novel extracellular PC-PLC/SMase toxin (i.e. PlcHR) of *P. aeruginosa*, using mammalian, EC-based in vitro angiogenesis assays and an in vivo vertebrate model (i.e. zebrafish). Even though there are other bacterial toxins, such as the Cytolethal Distending Toxins (CDTs), that are directly lethal to EC, and inhibit in vitro angiogenic processes (i.e. tube formation), they do not exhibit the degree of selectivity, we have observed with PlcHR [48]. CDTs are just as likely to be cytotoxic to an assortment of other cell types including: epithelial cells (HeLa, Hep-2), fibroblasts (human lung, human gingival) and immune effector cells (e.g. human macrophage, Jurket T-cells). Anthrax Lethal Toxin (ALT) has also been shown to: induce EC apoptosis, inhibit EC tube formation, and block the vascularization tumors in mice [49]–[51]. However, a more recent assessment of the effects of ALT in the context of an intact animal model, zebrafish, indicates that ALT does not cause significant EC cell death, while inhibiting angiogenesis [42]. Such data, indicate that, in vitro, as well as, in vivo models are necessary to fully and precisely elucidate the actual biological function of all antiangiogenic factors (e.g. ALT, PlcHR).

PlcH was initially called the “heat labile hemolysin” of *P. aeruginosa*. Early on it was shown to: (i) have modest cytotoxic effects on macrophage cell lines (unpublished observations) (ii) incite a strong chemokine response in mice and human granulocytes and (iii) profoundly suppress a PMA-stimulated neutrophil respiratory burst, at concentrations as low as 0.1 pg/ml [13],[23],[26]. However, no especially potent cytotoxic activity had been previously associated with PlcHR. What is more, its PC-PLC/SMase activity was presumed to be sufficient for its hemolytic or cytotoxic properties, through its ability to hydrolyse either PC or SM in the outer leaflet of eukaryotic cell membranes [13]. If that had indeed been the case, then based on the data presented herein, it would be problematical to reconcile that perception with the fact that not all cell lines examined (i.e. epithelial, fibroblasts, endothelial) are equally susceptible to PlcHR.

While they are asymmetrically distributed, the relative PC and SM content (i.e. ∼1∶1) in the outer leaflet of all mammalian cell lines tested in this study are nearly the same [29], and PlcHR is equally active on either type of phospholipid in micellar form (i.e. PC and SM) in vitro. However data in this report, provide plausible explanation for EC selectivity. Because RGD motifs of some microbial proteins (e.g. toxins, viral) bind to specific integrin receptors, it is not unreasonable to presume that PlcHR, through its RGD motif, interacts with specific integrin receptors on EC or CHO cells, which are not expressed by the other cells examined (HeLa, 1°CF lung epithelia). Its toxicity to EC however, still depends on its enzymatic activity, as clearly demonstrated by the lack of cytotoxicity of the Thr178Ala active site mutant to EC. Extraordinarily high concentrations (>1 µg/ml) of this mutant were required to exhibit any inhibition in the EC-dependent invasion assay and a 10-fold higher dose (i.e. 20 ng/embryo) of the mutant, compared to that used for the wild type PlcHR, produced no detectable phenotype in the zebrafish model.

With regard to the SMase activity of PlcHR, is of interest to note that a cyclic RGD (GRGDFL) peptide which blocks the function of αvβ3/αvβ5 integrin receptors, is currently being used in Phase III clinical trials as an antiangiogenic, antitumor, therapeutic agent named Cilengitide [52]–[54]. Cilengitide has been shown cause an increase in ceramide levels and induce EC apoptosis via a SMase-dependent mechanism [55],[56]. Perhaps, PlcHR, through its RGD motif and SMase activity that could very likely increase ceramide levels in EC, mimics the mechanism by which this antiangiogenic, antitumor therapeutic works. Since only picomolar (pM) concentrations of PlcHR are directly toxic to EC, then its mechanism of action is very likely associated with signaling events downstream of the hydrolysis of membrane PC or SM [33]. Although the generation of DAG from PC would be expected to induce EC proliferation, hydrolysis of membrane SM, by PlcHR, would cause a relatively increase in CM, likely leading to programmed cell death (i.e. apoptosis). In this regard treatment of EC with less than 2.5 ng/ml of PlcHR induced a significant increase in Caspase-3 activity, which was inhibitable by the pancaspase inhibitor Z-VAD-FMK (Figure 3 and Figure S2A and Figure S2B). Additionally, the type of calcium signaling observed with PlcHR (i.e. release of calcium from ER stores) is clearly associated with a SMase activity and not with PC-PLC activity [33]. On the other hand, it is still an enigma as to how PlcHR could raise the relative level of CM without affecting DAG levels, because it should be able to do both,. It may ultimately be possible to separate these activities, since PlcHR is hemolytic to sheep erythrocytes, which only contain sphingomyelin in the outer leaflet of their membranes. Likewise, it is of interest to note that the gene immediately 5′ to the *plcHR* operon encodes an extracellular ceramidase that could further contribute to EC death by producing sphingosine, a pro-apoptotic derivative of ceramide. Other scenarios are also possible, but further experimentation will be required to fully elucidate the mechanisms PlcHR lethality to EC.

Using the transparent zebrafish model, we also demonstrated the selectivity of PlcHR for endothelial cells, where dose-dependent cytotoxic effects can be monitored in real-time, independent of any of its unknown effects. In addition, the failure of the PlcHR-Thr178Ala to generate, in this animal model, any phenotypic changes at concentrations 10× higher those used for the wild type protein, persuasively corroborates our cellular assays (e.g. EC invasion assay) with this mutant. We have successfully defined a unique set of endothelial phenotypes and we have established a zebrafish model to further elucidate the mechanisms of the antiangiogenic effects of PlcHR. With regard to the use of this model in the context of *P. aeruginosa* infections there are now three recent reports describing the use of zebrafish to examine the virulence of *P. aeruginosa* [57]–[59]. A conclusion of one of these studies it that *P. aeruginosa* infection in zebrafish seems to best model an acute bacteremic infection associated with thermal injury or neutropenia. However, those observations do not necessarily exclude PlcHR from participating in the pathogenesis of chronic *P. aeruginosa* pulmonary infections. A recent study noted that the function of the cystic fibrosis transmembrane regulator (CFTR) is required for stress-induced apoptosis in lung endothelial cells by maintaining adequate CM activation [60]. These investigators suggested that aberrant endothelial cell death could disturb normal lung vascular homeostasis, thereby contributing to abnormal angiogenesis and to the chronic inflammation characteristically observed in CF patients. It would therefore not be surprising if the SMase of PlcHR produced by *P. aeruginosa* also contributes to apoptosis in lung endothelial cells and further exacerbates chronic inflammation seen in CF patients.

PlcHR is a complex heterodimeric protein, with several distinctive features, which presently have no known functions (Figure S3). For example, nearly all of the PLC members of this large family carry duplicate, C-terminal domains of unknown function, comprising nearly one third of their lengths. Additionally, even though it is understood that PlcR is a chaperone required for the secretion of PlcH through the inner membrane via the twin arginine translocase (TAT) secretory system and through the outer membrane via the Xcp secretory system of *P. aeruginosa* [61]–[63], its contributions to the properties of PlcHR investigated in this report, are not entirely certain. It is very likely that the sensitivity and tractability of the zebrafish assay, along with site-specific mutagenesis of these intriguing regions of PlcH, will reveal insights into their biological roles (e.g. trafficking in a whole animal) that might not otherwise be revealed through conventional in vitro assays (e.g. enzymatic, cytotoxicity to cultured EC). Moreover, the versatility of the zebrafish model for chemical biology, genetics and targeted gene knockdown by morpholinos [64] suggests that future studies on host components (e.g. specific integrin receptors, signaling mechanisms downstream of CM) required for PlcHR's endothelial toxicity could be investigated using this inexpensive vertebrate model. For example, a downstream derivative of CM and sphingosine, sphingosine-1-phosphate is currently being investigated as a therapeutic based on its antiapoptotic properties [65].

Data presented herein, provide some additional insights into the role that PlcHR might play in particular types of infection caused by *P. aeruginosa*. During septicemia, this opportunist has a proclivity to invade blood vessels, thereby inciting vasculitis and causing vascular necrosis. However, bacterial invasion of the vasculature is not necessarily found near or in the areas of necrosis, and organisms are not found in all necrotic vessel walls. It has been suggested however, that the toxic products expressed by *P. aeruginosa* (e.g. PlcHR, Exotoxin A) most likely play a role in this type of organism-distal vascular pathology [64]. The effects of PlcHR on EC, in vitro angiogenesis, and in zebrafish are therefore entirely consistent with this aspect of the pathogenesis of *P. aeruginosa* sepsis.

Finally, Anthrax toxin and some of its variants have been examined for their ability, through their antiangiogenic properties, to block the vascularization and growth of tumors implanted in animal models [66],[67]. Accordingly, it is not unreasonable to suggest that it would be worthwhile to further investigate the ability of PlcHR to inhibit angiogenesis, related to tumor growth, as well as in eye diseases where angiogenesis directly contributes to pathogenesis (e.g. macular degeneration). While, there may be concerns about the use of bacterial toxins as therapeutic agents, some of which are based on their immunogenicity, this concern may be mitigated for therapeutic applications in the eye, which is considered to be an immunologically privileged site [68].

## Materials and Methods

### Enzyme assays

PC-PLC and SMase assays were performed as previously described [13].

### Production and purification of PlcHR wild type, a PlcH Thr179Ala mutant, and RGD mutants

Production and purification of PlcHR and PlcH from *P. aeruginosa* were performed as previously described [13]. The Thr178Ala *plcH* mutant was identified from an alanine scanning mutagenesis experiment to identify enzymatically deficient PlcH. A region containing the segment of the *plcH* gene that encodes the Thr178Ala mutation was then used to replace the corresponding *plcH* wild type sequence in the *P. aeruginosa* PlcHR expression plasmid with a T7 promoter [13]. The mutant *plcH* gene along with the wild type *plcR* gene were expressed in the same *P. aeruginosa* T7 expression system that is used for expression of wild type PlcHR [13]. The mutant (i.e. Thr178Ala) was purified using the same protocol for wild type PlcHR [13] except that fractions recovered during purification, which contained the Thr178Ala PlcHR mutant were detected using monoclonal antibodies against PlcH and PlcR [13]. RGD mutants were constructed by site-specific mutagenesis methods as previously described. In each case the mutant PlcH gene was expressed in the *P. aeruginosa* T7 expression system along with the wild type *plcR* gene.

### Cytotoxicity assays and PlcHR binding assay

In all cases in this report where endothelial cells (EC) are used they are: Human Umbilical Vascular Endothelial Cells (HUVEC). They were acquired from BD biosciences (San Jose, CA). All primary cell lines (e.g. HUVEC, CF lung epithelial) were always used at less than 8 passages. Assays were performed with the designated cell lines according to manufacturer's recommendation using the CytoTox 96 Assay Kit (Promega Corporation). Percent activity was determined by performing a total lysis control in which cells were lysed with 1% triton X-100. Percent lysis was determined via the formula. Percent lysis = [(experimental−spontaneous control)/(Total lysis−spontaneous control)]×100 To measure binding of PlcHR to CHO cells (Figure 5B) were grown in 24 well tissue culture dishes until they reached 80–90% confluence. The media was replaced with 300 µl of media containing 7.5 ng/ml wild type or a mutant PlcHR. The cells were incubated for 2 h at 37°C with 5% CO_2_. Control samples consisted of 7.5 ng/ml mutant or wild type PlcHR_2_ incubated in 300 µl media alone. To determine the percent of activity (enzyme) bound by the cells, the PLC activity in the supernatants from the wild type or mutant PlcHR samples incubated with cells was compared to the control samples. PLC activity was detected using the synthetic substrate ρ-nitrophenyl-phosphorylcholine as previously described [13], which detects PlcHR activity, but it does not react at all with CHO cells that are not exposed to PlcHR. The difference in recovered activity between the controls and the samples with cells was then used to calculate the percent of activity bound to the cells. Percent activity bound = [(control activity - activity with cells)/control activity]*100. Lysing the cells with 1% Triton X-100 recovered all activity.

### Caspase-3 activation assay

Caspase-3 activity was assayed with the colorimetric CaspACE Assay system (Promega, Madison, WI). The colorimetric substrate (Ac-DEVD-pNA) is hydrolyzed by activated caspase-3 releasing pNA producing a yellow color that is quantified at an absorbance of 405 nm. Camptothecin, a topoisomerase I inhibitor, was used as a positive control for activation of caspase-3 and induction of apoptosis. For inhibition of apoptosis ZVAD- FMK was added to samples at a final concentration of 50 µM. HUVEC were cultivated in 6 well tissue culture dishes to 80–90% confluency at which time the media was exchanged with 2 ml of fresh media containing PlcHR2 or other compounds to be examined. The cultures were allowed to incubate at 37°C in 5% CO2 for 3 to 16 hours. The cells were harvested by trypsin/EDTA treatment, washed with ice cold PBS and suspended in lysis buffer at a concentration of 1×108 cells/ml. To prepare lysates cells were freeze-thawed four times and sonicated twice for 15 seconds at level 10 in a Sonic Dismembrator Model 100 (Fisher Scientific, Hampton, NH). The lysates were incubated on ice for 15 minutes before the cell lysate supernatant was harvested by centrifuge at 16,100×g for 20 minutes at 4°C. Caspase-3 activity in the cell lysates was assayed for by the manufactures' recommended protocol.

### Angiogenesis assays

EC migration assays, EC invasion assays and EC tube formation assays were performed according to manufacturer's recommendations using the BD Biocoat Angiogenesis System (BD Biosciences) with primary cultures of HUVEC.

### Animals

All animal protocols were approved by the Institutional Animal Care and Use Committee of Children's Hospital Boston. Breeding fish, wild type (AB strain), or transgenic lines, were maintained at 28.5°C on a 14 h light/10 h dark cycle. Embryos were collected by natural spawning, and raised in 10% Hank's saline at 32°C.

### Microinjection of toxin proteins and inhibitors

Microinjections into the zebrafish vasculature at 48 hpf were carried out as described by Bolcome et al. (2008). PlcHR or PlcHR-Thr178Ala were diluted immediately before, and kept at 4°C until injection. Injected amounts are indicated in the figure legends for each experiment. Phenol red (0.05%) was added to each condition for visibility during microinjection. Volumes of 40 nl or less were delivered into the common cardinal vein of embryos anesthetized with tricaine (Sigma) at 48 hpf using a gas driven microinjector (Medical Systems Corp.). After injection, embryos were transferred into fresh medium for recovery, maintained at 32°C, and scored for toxin action at time points indicated in the text.

### Acridine orange staining

Dechorionated embryos were placed in 5 mL of a 50 µg/mL solution of acridine orange (acridinium chloride hemi-[zinc chloride], Sigma) in 10% Hank's saline solution at room temperature. After 30 minutes of staining while protected from light, embryos were washed three times for 10 minutes with 10% Hank's saline. Following the third wash, embryos were mounted on glass slides for fluorescence microscopy.

## Supporting Information

Figure S1 A detailed analysis of PlcHR cytotoxicity of two susceptible (HUVEC, CHO) and two resistant cell lines (L929 and Hela). Time and dose killing of various cell types. The cells indicated were treated with increasing concentrations of PlcHR for increasing lengths of time. The arrows indicate increasing time of treatment. HUVECs, CHO, and L929 were treated for 2, 4, 6, 8, 10, and 22 h, and the HeLa cells were treated for 3, 6, 9, 12, and 22 h.
(1.41 MB TIF)

Figure S2 Effect of PlcHR on caspase-3 expression. (A) The pan-caspase inhibitor Z-VAD-FMK completely inhibits PlcHR activation of caspase-3 and reduced the level of LDH release in HUVECs. Caspase-3 activation and LDH release were measured at 16 h post-treatment of HUVEC with 2.5 ng PlcHR. Z-VAD-FMK is a potent, irreversible, and cell-permeable pan-caspase inhibitor. (B) PlcHR activates caspase-3 between 3 and 6 h. HUVEC were treated with 2.5 ng/ml PlcHR2 for 3, 6, and 9 h. At each time point, caspase-3 activity was assayed as described in Materials and Methods.
(0.21 MB TIF)

Figure S3 Key structural features of PlcHR and members of the Phosphodiester/PLC Superfamily. PlcH is the only known member of this family to be associated with a PlcR-like protein.
(9.25 MB TIF)

Table S1 Inhibition of EC tube formation by PlcHR. Pre-tube formation: Endothelial cells were challenged with 4–64 ng/ml during tube formation (24 h) on matrigel. Post-tube formation: Endothelial cells that had already formed tubes after 48 h were then challenged with 4–64 ng/ml of PlcHR for an additional 20 h. Photographs were taken at 40× magnification. Tube length was measured with Metamorph Ver 7.1.6.0 software (Molecular Devices, Sunnyvale, CA).
(0.04 MB DOC)

## References

[1] Clarke CJ, Snook CF, Tani M, Matmati N, Marchesini N (2006). The extended family of neutral sphingomyelinases.. Biochemistry.

[2] Glunde K, Serkova NJ (2006). Therapeutic targets and biomarkers identified in cancer choline phospholipid metabolism.. Pharmacogenomics.

[3] Whatley RE, Zimmerman GA, McIntyre TM, Prescott SM (1990). Lipid metabolism and signal transduction in endothelial cells.. Prog Lipid Res.

[4] Teichgraber V, Ulrich M, Endlich N, Riethmuller J, Wilker B (2008). Ceramide accumulation mediates inflammation, cell death and infection susceptibility in cystic fibrosis.. Nat Med.

[5] Yu H, Zeidan YH, Wu BX, Jenkins RW, Flotte TR (2009). Defective Acid Sphingomyelinase Pathway with *Pseudomonas aeruginosa* Infection in Cystic Fibrosis.. Am J Respir Cell Mol Biol. e-pub ahead of print January 23, 2009.

[6] Titball RW (1998). Bacterial phospholipases.. Symp Ser Soc Appl Microbiol.

[7] Titball RW, Naylor CE, Basak AK (1999). The *Clostridium perfringens* alpha-toxin.. Anaerobe.

[8] Smith GA, Marquis H, Jones S, Johnston NC, Portnoy DA (1995). The two distinct phospholipases C of *Listeria monocytogenes* have overlapping roles in escape from a vacuole and cell-to-cell spread.. Infect Immun.

[9] Martin SF, Follows BC, Hergenrother PJ, Trotter BK (2000). The choline binding site of phospholipase C (*Bacillus cereus*): Insights into substrate specificity.. Biochemistry.

[10] Alape-Giron A, Flores-Diaz M, Guillouard I, Naylor CE, Titball RW (2000). Identification of residues critical for toxicity in *Clostridium perfringens* phospholipase C, the key toxin in gas gangrene.. Eur J Biochem.

[11] Titball RW, Fearn AM, Williamson ED (1993). Biochemical and immunological properties of the C-terminal domain of the alpha-toxin of *Clostridium perfringens*.. FEMS Microbiol Lett.

[12] Titball RW, Leslie DL, Harvey S, Kelly D (1991). Hemolytic and sphingomyelinase activities of *Clostridium perfringens* alpha-toxin are dependent on a domain homologous to that of an enzyme from the human arachidonic acid pathway.. Infect Immun.

[13] Stonehouse MJ, Cota-Gomez A, Parker SK, Martin WE, Hankin JA (2002). A novel class of microbial phosphocholine-specific phospholipases C.. Mol Microbiol.

[14] Felts RL, Reilly TJ, Tanner JJ (2006). Structure of *Francisella tularensis* AcpA: Prototype of a unique superfamily of acid phosphatases and phospholipases C.. J Biol Chem.

[15] Korbsrisate S, Tomaras AP, Damnin S, Ckumdee J, Srinon V (2007). Characterization of two distinct phospholipase C enzymes from *Burkholderia pseudomallei*.. Microbiology.

[16] Raynaud C, Guilhot C, Rauzier J, Bordat Y, Pelicic V (2002). Phospholipases C are involved in the virulence of *Mycobacterium tuberculosis*.. Mol Microbiol.

[17] Viana-Niero C, de Haas PE, van Soolingen D, Leao SC (2004). Analysis of genetic polymorphisms affecting the four phospholipase C (plc) genes in *Mycobacterium tuberculosis* complex clinical isolates.. Microbiology.

[18] Yang Z, Yang D, Kong Y, Zhang L, Marrs CF (2005). Clinical relevance of *Mycobacterium tuberculosis plcD* gene mutations.. Am J Respir Crit Care Med.

[19] Ostroff RM, Wretlind B, Vasil ML (1989). Mutations in the hemolytic-phospholipase C operon result in decreased virulence of *Pseudomonas aeruginosa* PAO1 grown under phosphate-limiting conditions.. Infect Immun.

[20] Rahme LG, Stevens EJ, Wolfort SF, Shao J, Tompkins RG (1995). Common virulence factors for bacterial pathogenicity in plants and animals.. Science.

[21] Hogan DA, Kolter R (2002). Pseudomonas-Candida interactions: An ecological role for virulence factors.. Science.

[22] Montes LR, Lopez DJ, Sot J, Bagatolli LA, Stonehouse MJ (2008). Ceramide-enriched membrane domains in red blood cells and the mechanism of sphingomyelinase-induced hot-cold hemolysis.. Biochemistry.

[23] Montes LR, Ibarguren M, Goni FM, Stonehouse M, Vasil ML (2007). Leakage-free membrane fusion induced by the hydrolytic activity of PlcHR(2), a novel phospholipase C/sphingomyelinase from *Pseudomonas aeruginosa*.. Biochim Biophys Acta.

[24] Soave R, Murray HW, Litrenta MM (1978). Bacterial invasion of pulmonary vessels. Pseudomonas bacteremia mimicking pulmonary thromboembolism with infarction.. Am J Med.

[25] Ziegler EJ, Douglas H (1979). *Pseudomonas aeruginosa* vasculitis and bacteremia following conjunctivitis: A simple model of fatal pseudomonas infection in neutropenia.. J Infect Dis.

[26] Terada LS, Johansen KA, Nowbar S, Vasil AI, Vasil ML (1999). *Pseudomonas aeruginosa* hemolytic phospholipase C suppresses neutrophil respiratory burst activity.. Infect Immun.

[27] Wieland CW, Siegmund B, Senaldi G, Vasil ML, Dinarello CA (2002). Pulmonary inflammation induced by *Pseudomonas aeruginosa* lipopolysaccharide, phospholipase C, and exotoxin A: Role of interferon regulatory factor 1.. Infect Immun.

[28] Zabner J, Karp P, Seiler M, Phillips SL, Mitchell CJ (2003). Development of cystic fibrosis and noncystic fibrosis airway cell lines.. Am J Physiol Lung Cell Mol Physiol.

[29] Alberts B, Johnson A, Lewis J, Raff M, Roberts K (2002). The Molecular Biology of the Cell. 4th ed.

[30] Wadsworth SJ, Goldfine H (1999). *Listeria monocytogenes* phospholipase C-dependent calcium signaling modulates bacterial entry into J774 macrophage-like cells.. Infect Immun.

[31] Wadsworth SJ, Goldfine H (2002). Mobilization of protein kinase C in macrophages induced by *Listeria monocytogenes* affects its internalization and escape from the phagosome.. Infect Immun.

[32] Pinton P, Ferrari D, Rapizzi E, Di Virgilio F, Pozzan T (2001). The Ca2+ concentration of the endoplasmic reticulum is a key determinant of ceramide-induced apoptosis: Significance for the molecular mechanism of Bcl-2 action.. EMBO J.

[33] Hannun YA, Obeid LM (2008). Principles of bioactive lipid signalling: Lessons from sphingolipids.. Nat Rev Mol Cell Biol.

[34] Boatright KM, Salvesen GS (2003). Mechanisms of caspase activation.. Curr Opin Cell Biol.

[35] Takada Y, Ye X, Simon S (2007). The integrins.. Genome Biol.

[36] Assa-Munt N, Jia X, Laakkonen P, Ruoslahti E (2001). Solution structures and integrin binding activities of an RGD peptide with two isomers.. Biochemistry.

[37] van der Most RG, Corver J, Strauss JH (1999). Mutagenesis of the RGD motif in the yellow fever virus 17D envelope protein.. Virology.

[38] Zhao Q, Pacheco JM, Mason PW (2003). Evaluation of genetically engineered derivatives of a Chinese strain of foot-and-mouth disease virus reveals a novel cell-binding site which functions in cell culture and in animals.. J Virol.

[39] Staton CA, Lewis C, Bricknell R (2006). Angiogenesis Assays: A critical appraisal of current techniques. 1st ed.

[40] Mohapatra NP, Balagopal A, Soni S, Schlesinger LS, Gunn JS (2007). AcpA is a Francisella acid phosphatase that affects intramacrophage survival and virulence.. Infect Immun.

[41] Reilly TJ, Baron GS, Nano FE, Kuhlenschmidt MS (1996). Characterization and sequencing of a respiratory burst-inhibiting acid phosphatase from *Francisella tularensis*.. J Biol Chem.

[42] Bolcome RE, Sullivan SE, Zeller R, Barker AP, Collier RJ (2008). Anthrax lethal toxin induces cell death-independent permeability in zebrafish vasculature.. Proc Natl Acad Sci U S A.

[43] Isogai S, Horiguchi M, Weinstein BM (2001). The vascular anatomy of the developing zebrafish: An atlas of embryonic and early larval development.. Dev Biol.

[44] Lawson ND, Weinstein BM (2002). In vivo imaging of embryonic vascular development using transgenic zebrafish.. Dev Biol.

[45] Traver D, Paw BH, Poss KD, Penberthy WT, Lin S (2003). Transplantation and in vivo imaging of multilineage engraftment in zebrafish bloodless mutants.. Nat Immunol.

[46] Roman BL, Pham VN, Lawson ND, Kulik M, Childs S (2002). Disruption of acvrl1 increases endothelial cell number in zebrafish cranial vessels.. Development.

[47] Galloway JL, Wingert RA, Thisse C, Thisse B, Zon LI (2005). Loss of gata1 but not gata2 converts erythropoiesis to myelopoiesis in zebrafish embryos.. Dev Cell.

[48] Smith JL, Bayles DO (2006). The contribution of cytolethal distending toxin to bacterial pathogenesis.. Crit Rev Microbiol.

[49] Abi-Habib RJ, Singh R, Leppla SH, Greene JJ, Ding Y (2006). Systemic anthrax lethal toxin therapy produces regressions of subcutaneous human melanoma tumors in athymic nude mice.. Clin Cancer Res.

[50] Kirby JE (2004). Anthrax lethal toxin induces human endothelial cell apoptosis.. Infect Immun.

[51] Rogers MS, Christensen KA, Birsner AE, Short SM, Wigelsworth DJ (2007). Mutant anthrax toxin B moiety (protective antigen) inhibits angiogenesis and tumor growth.. Cancer Res.

[52] MacDonald TJ, Stewart CF, Kocak M, Goldman S, Ellenbogen RG (2008). Phase I clinical trial of cilengitide in children with refractory brain tumors: Pediatric Brain Tumor Consortium Study PBTC-012.. J Clin Oncol.

[53] Reardon DA, Nabors LB, Stupp R, Mikkelsen T (2008). Cilengitide: An integrin-targeting arginine-glycine-aspartic acid peptide with promising activity for glioblastoma multiforme.. Expert Opin Investig Drugs.

[54] Smith JW (2003). Cilengitide Merck.. Curr Opin Investig Drugs.

[55] Avraamides CJ, Garmy-Susini B, Varner JA (2008). Integrins in angiogenesis and lymphangiogenesis.. Nat Rev Cancer.

[56] Erdreich-Epstein A, Tran LB, Cox OT, Huang EY, Laug WE (2005). Endothelial apoptosis induced by inhibition of integrins alphavbeta3 and alphavbeta5 involves ceramide metabolic pathways.. Blood.

[57] Brannon MK, Davis JM, Mathias JR, Hall CJ, Emerson JC (2009). Pseudomonas aeruginosa Type III secretion system interacts with phagocytes to modulate systemic infection of zebrafish embryos.. Cell Microbiol.

[58] Clatworthy AE, Lee JS, Leibman M, Kostun Z, Davidson AJ (2009). Pseudomonas aeruginosa infection of zebrafish involves both host and pathogen determinants.. Infect Immun.

[59] Rawls JF, Mahowald MA, Goodman AL, Trent CM, Gordon JI (2007). In vivo imaging and genetic analysis link bacterial motility and symbiosis in the zebrafish gut.. Proc Natl Acad Sci U S A.

[60] Noe J, Petrusca D, Rush N, Deng P, Vandemark M (2009). CFTR Regulation of Intracellular pH and Ceramides is Required for Lung Endothelial Cell Apoptosis.. Am J Respir Cell Mol Biol [January 23, 2009, e-pub ahead of print].

[61] Cota-Gomez A, Vasil AI, Kadurugamuwa J, Beveridge TJ, Schweizer HP (1997). PlcR1 and PlcR2 are putative calcium-binding proteins required for secretion of the hemolytic phospholipase C of Pseudomonas aeruginosa.. Infect Immun.

[62] Ochsner UA, Snyder A, Vasil AI, Vasil ML (2002). Effects of the twin-arginine translocase on secretion of virulence factors, stress response, and pathogenesis.. Proc Natl Acad Sci U S A.

[63] Snyder A, Vasil AI, Zajdowicz SL, Wilson ZR, Vasil ML (2006). Role of the *Pseudomonas aeruginosa* PlcH Tat signal peptide in protein secretion, transcription, and cross-species Tat secretion system compatibility.. J Bacteriol.

[64] Lieschke GJ, Currie PD (2007). Animal models of human disease: Zebrafish swim into view.. Nat Rev Genet.

[65] Takabe K, Paugh SW, Milstien S, Spiegel S (2008). “Inside-out” signaling of sphingosine-1-phosphate: Therapeutic targets.. Pharmacol Rev.

[66] Liu S, Wang H, Currie BM, Molinolo A, Leung HJ (2008). Matrix metalloproteinase-activated anthrax lethal toxin demonstrates high potency in targeting tumor vasculature.. J Biol Chem.

[67] Alfano RW, Leppla SH, Liu S, Bugge TH, Duesbery NS (2008). Potent inhibition of tumor angiogenesis by the matrix metalloproteinase-activated anthrax lethal toxin: Implications for broad anti-tumor efficacy.. Cell Cycle.

[68] Ferguson TA, Green DR, Griffith TS (2002). Cell death and immune privilege.. Int Rev Immunol.

