# Supplementary figures and images for: A Complex Extracellular Sphingomyelinase of Pseudomonas aeruginosa Inhibits Angiogenesis by Selective Cytotoxicity to Endothelial Cells
Source: PLoS Pathog. 2009 May 8;5(5):e1000420. doi: 10.1371/journal.ppat.1000420 (PMC2673038; doi:10.1371/journal.ppat.1000420)

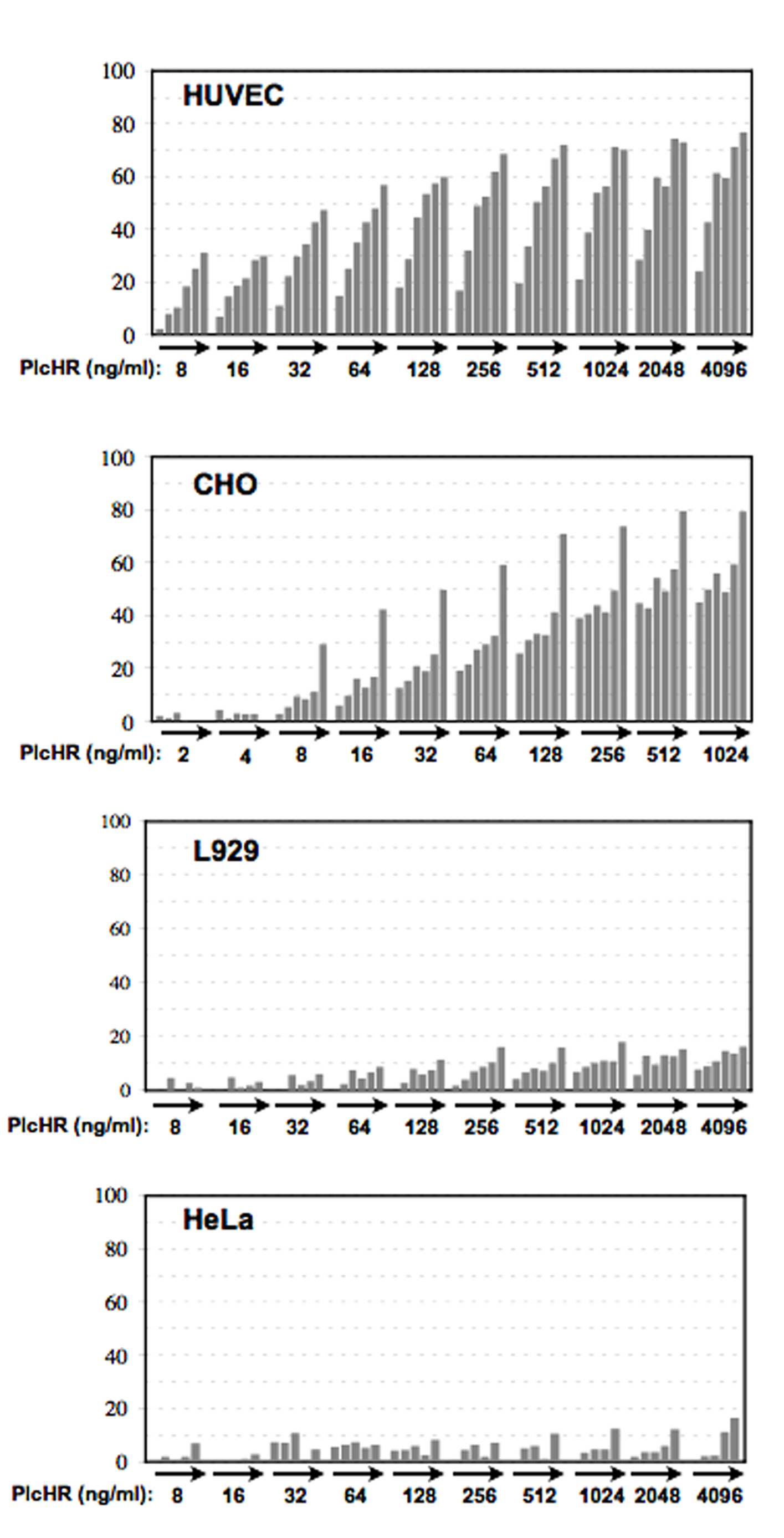

Supplement: Figure S1 — A detailed analysis of PlcHR cytotoxicity of two susceptible (HUVEC, CHO) and two resistant cell lines (L929 and Hela). Time and dose killing of various cell types. The cells indicated were treated with increasing concentrations of PlcHR for increasing lengths of time. The arrows indicate increasing time of treatment. HUVECs, CHO, and L929 were treated for 2, 4, 6, 8, 10, and 22 h, and the HeLa cells were treated for 3, 6, 9, 12, and 22 h. (1.41 MB TIF) [file ppat.1000420.s001.tif]

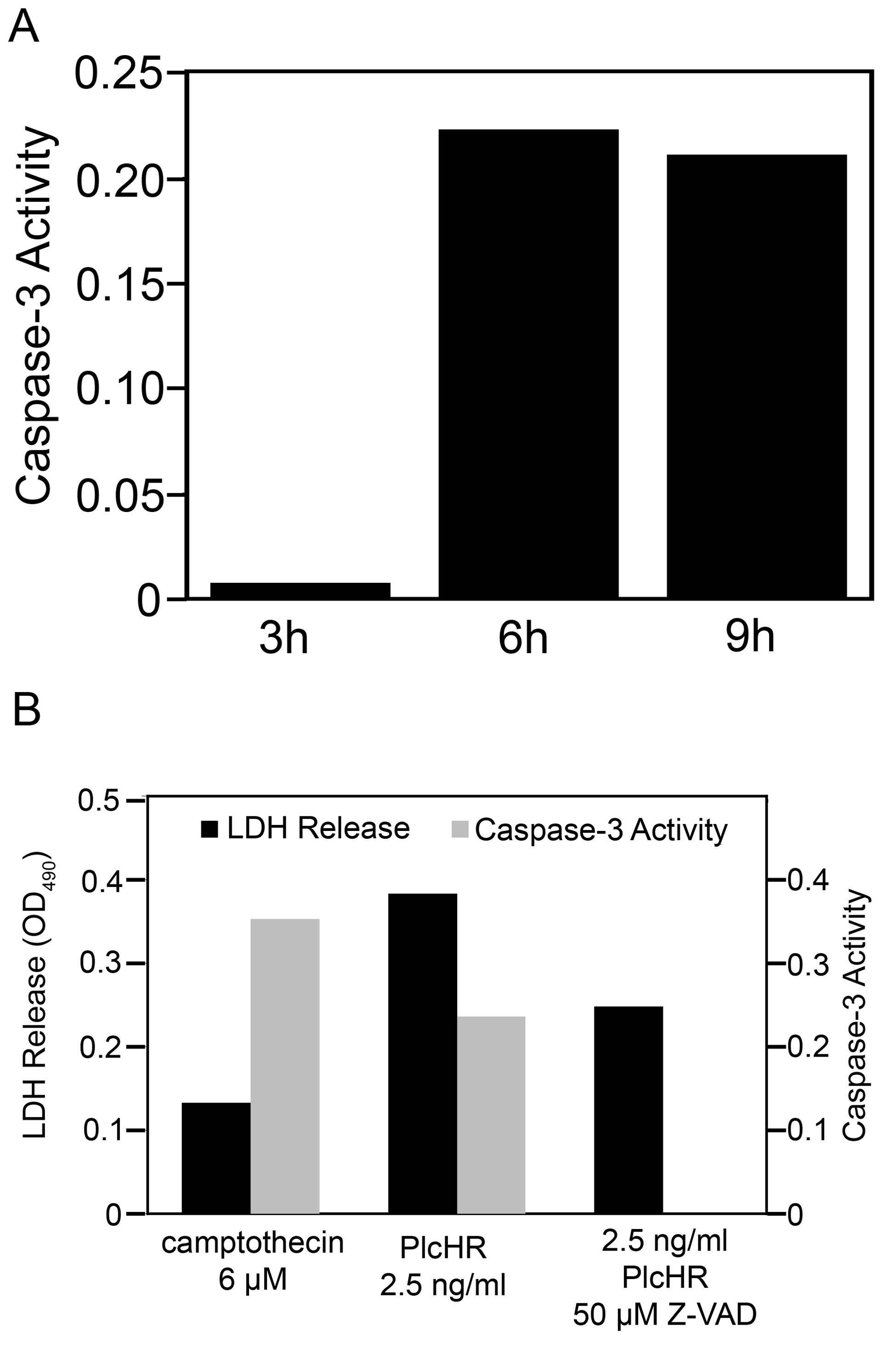

Supplement: Figure S2 — Effect of PlcHR on caspase-3 expression. (A) The pan-caspase inhibitor Z-VAD-FMK completely inhibits PlcHR activation of caspase-3 and reduced the level of LDH release in HUVECs. Caspase-3 activation and LDH release were measured at 16 h post-treatment of HUVEC with 2.5 ng PlcHR. Z-VAD-FMK is a potent, irreversible, and cell-permeable pan-caspase inhibitor. (B) PlcHR activates caspase-3 between 3 and 6 h. HUVEC were treated with 2.5 ng/ml PlcHR2 for 3, 6, and 9 h. At each time point, caspase-3 activity was assayed as described in Materials and Methods. (0.21 MB TIF) [file ppat.1000420.s002.tif]

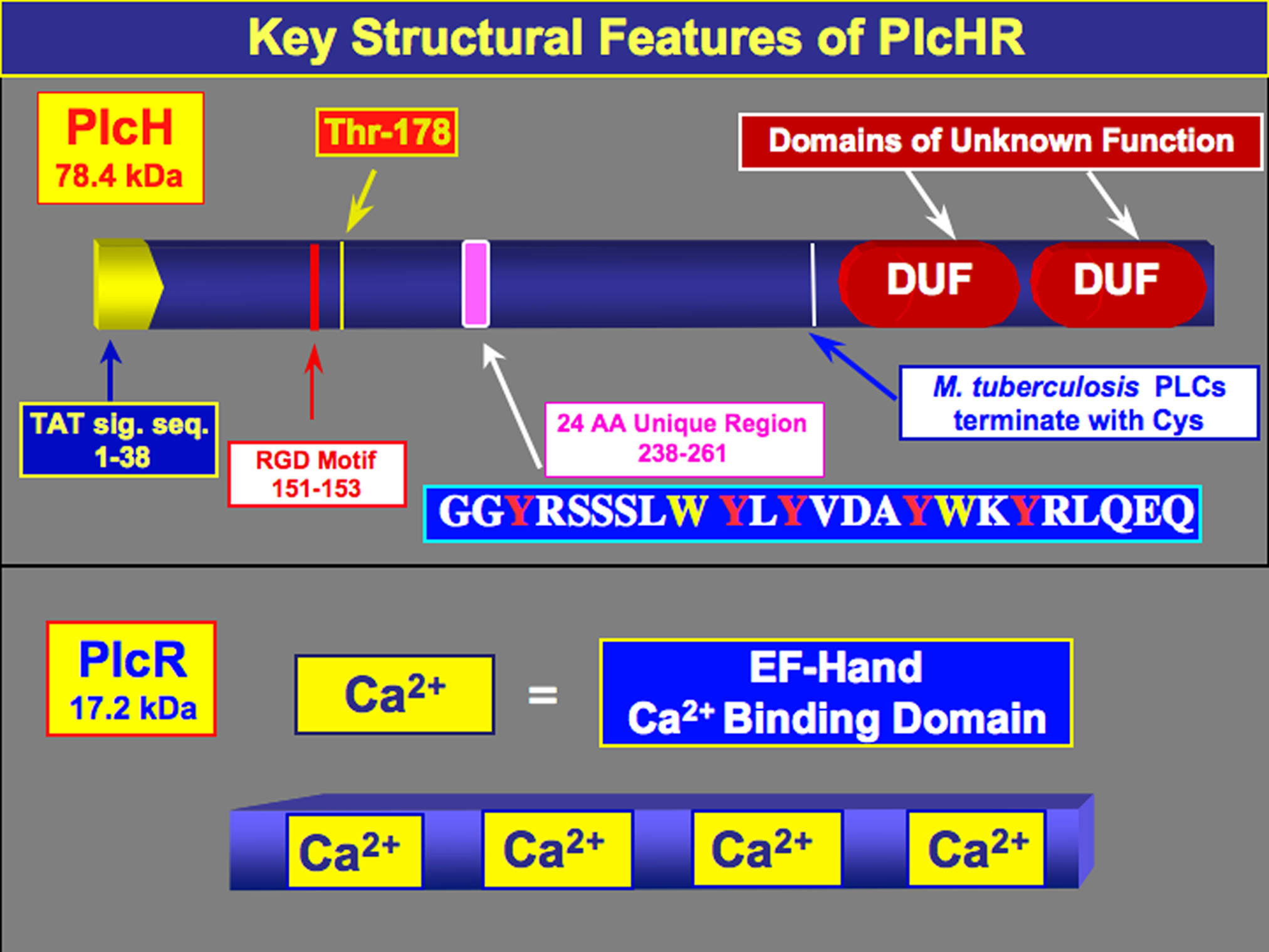

Supplement: Figure S3 — Key structural features of PlcHR and members of the Phosphodiester/PLC Superfamily. PlcH is the only known member of this family to be associated with a PlcR-like protein. (9.25 MB TIF) [file ppat.1000420.s003.tif]
