# Supplementary material for: A Complex Extracellular Sphingomyelinase of Pseudomonas aeruginosa Inhibits Angiogenesis by Selective Cytotoxicity to Endothelial Cells
Source: PLoS Pathog. 2009 May 8;5(5):e1000420. doi: 10.1371/journal.ppat.1000420 (PMC2673038; doi:10.1371/journal.ppat.1000420)
Supplement: Table S1 — Inhibition of EC tube formation by PlcHR. Pre-tube formation: Endothelial cells were challenged with 4–64 ng/ml during tube formation (24 h) on matrigel. Post-tube formation: Endothelial cells that had already formed tubes after 48 h were then challenged with 4–64 ng/ml of PlcHR for an additional 20 h. Photographs were taken at 40× magnification. Tube length was measured with Metamorph Ver 7.1.6.0 software (Molecular Devices, Sunnyvale, CA). (0.04 MB DOC) [file ppat.1000420.s004.doc]

**Table S1. Inhibition of EC tube formation by PlcHR**

|  | Pre-Tube Formation | | Post Tube Formation | |
| --- | --- | --- | --- | --- |
| Sample | Total Tube Lenght | Avg. Tube Length | Total Tuber Length | Avg. Tube Lenght |
| 0 ng/ml | 8682 | 105 | 5644 | 123 |
| 4 ng/ml | 3231 | 60 | 9328 | 121 |
| 8 ng/ml | 2007 | 39 | 7444 | 128 |
| 16 ng/ml | 2168 | 43 | 7140 | 143 |
| 32 ng/ml | 2103 | 50 | 2729 | 78 |
| 64 ng/ml | 2076 | 40 | 2786 | 93 |

Pre-tube Formation: Endothelial cells were challenged with 4-64 ng/ml during tube formation (24 hrs) on matrigel. Post Tube Formation: Endothelial cells that had already formed tubes after 48 hrs were then challenged with 4-64 ng/ml of PlcHR for an additional 20 hrs. Photographs were taken at 40X magnification. Tube length was measured with Metamorph Ver 7.1.6.0 software (Molecular Devices, Sunnyvale, CA).
